# Machine Learning-Based Prediction of Ultrasound-Detected Hepatic Steatosis Within the Metabolic Dysfunction-Associated Steatotic Liver Disease Spectrum Using Routine Clinical and Biochemical Parameters

**DOI:** 10.3390/biomedicines14051154

**Published:** 2026-05-20

**Authors:** Canan Akkus, Gamze Sonmez, Ali Sahin, Yigit Yazarkan, Melis Gokgoz, Feride Caglar, Sanem Kayhan

**Affiliations:** 1 The Department of Internal Medicine, Ankara Etlik City Hospital, Ankara 06170, Türkiye; drmelisgokgoz@gmail.com (M.G.); drferidecaglar@gmail.com (F.C.); drkayhansanem@yahoo.com (S.K.); 2 Faculty of Medicine, Hacettepe University, Ankara 06230, Türkiye; gsonmez1999@gmail.com (G.S.); yyazarkan99@gmail.com (Y.Y.); 3 Department of Emergency Service, Dr. Vefa Tanır Ilgın State Hospital, Konya 42600, Türkiye; sahin@silicosome.com

**Keywords:** metabolic dysfunction-associated steatotic liver disease, machine learning, logistic regression, hepatic steatosis, predictive modeling

## Abstract

**Background/Objectives:** Metabolic dysfunction-associated steatotic liver disease (MASLD) is now the leading cause of chronic liver disease globally, mirroring the increasing prevalence of obesity, insulin resistance, and type 2 diabetes. Early detection of hepatic steatosis is vital for cardiometabolic risk assessment; however, conventional imaging is costly and impractical for population screening. This study aimed to develop interpretable machine-learning models to predict ultrasound-detected hepatic steatosis within the MASLD spectrum using routinely available clinical and biochemical data. **Methods:** We analyzed data from 644 adults, 50% of whom had ultrasound-detected hepatic steatosis. Preprocessing, imputation, and feature selection were implemented within a single scikit-learn pipeline to avoid information leakage. An Elastic Net-regularized logistic regression identified the top 20 predictors, which were subsequently used across nine supervised machine learning (ML) classifiers. Model performance was evaluated via repeated stratified 5-fold cross-validation (25 resamples) using accuracy, F1 score, sensitivity, specificity, Youden’s J, balanced accuracy, and Area Under the Receiver Operating Characteristic Curve (AUROC). Interpretability was assessed using SHapley Additive exPlanations (SHAP). **Results:** Participants with ultrasound-detected hepatic steatosis exhibited greater adiposity, insulin resistance, and dyslipidemia compared with controls [*p* < 0.05 for body mass index (BMI), waist circumference, glucose, glycated hemoglobin (HbA1c), triglycerides]. Elastic Net selection highlighted Weight, Ponderal Index, Fibrosis-4 Index (FIB-4), blood urea nitrogen (BUN)/Creatinine ratio, Aspartate Aminotransferase to Platelet Ratio Index (APRI), and Visceral Adiposity Index as the strongest predictors. Logistic Regression and Gradient Boosting achieved the best performance (accuracy = 0.65 ± 0.03; AUROC = 0.71 ± 0.04; balanced accuracy = 0.66 ± 0.06), outperforming rule-based indices such as Fatty Liver Index (FLI) and Hepatic Steatosis Index (HSI) reported in the literature. SHAP analysis confirmed clinically coherent feature effects, with higher anthropometric and hepatic injury indices increasing the predicted probability of ultrasound-detected hepatic steatosis. **Conclusions:** Routinely available clinical and biochemical parameters can predict hepatic steatosis with moderate accuracy using transparent, interpretable ML models. Logistic Regression and Gradient Boosting provided best discrimination and robust internal performance, offering a pragmatic, low-cost approach for early identification of ultrasound-detected hepatic steatosis within the MASLD spectrum in primary and metabolic care settings.

## 1. Introduction

Metabolic dysfunction-associated steatotic liver disease (MASLD) has emerged as the most prevalent chronic liver disorder worldwide, paralleling the global epidemics of obesity, insulin resistance, and type 2 diabetes mellitus [1,2]. Hepatic steatosis represents the earliest and most common manifestation of MASLD and is a key determinant of progression to steatohepatitis, fibrosis, cirrhosis, and hepatocellular carcinoma. Beyond liver-related outcomes, steatosis is also strongly associated with increased cardiometabolic risk and all-cause mortality [3,4]. Therefore, early and scalable identification of hepatic steatosis is essential for effective risk stratification and preventive interventions.

Current diagnostic strategies rely predominantly on imaging modalities, including ultrasonography, controlled attenuation parameter (CAP), and magnetic resonance imaging–proton density fat fraction (MRI-PDFF), or, in selected cases, liver biopsy [5,6]. Although these methods provide reasonable diagnostic accuracy, their cost, limited accessibility, operator dependence, and, in the case of biopsy, invasiveness restrict their applicability for large-scale screening and routine use in primary care settings.

To overcome these limitations, simple clinical indices such as the fatty liver index (FLI) and hepatic steatosis index (HSI) have been developed using conventional regression-based approaches [7,8]. While attractive for their simplicity, these scores are constrained by predefined linear assumptions, limited variable interactions, and reduced performance across heterogeneous populations and metabolic phenotypes. As MASLD is a multifactorial and metabolically complex disease, such simplifications may fail to capture the nonlinear and synergistic relationships among routinely collected clinical and laboratory variables.

Recent advances in machine learning (ML) provide new opportunities to address these limitations. ML algorithms can integrate high-dimensional clinical data and model complex, nonlinear interactions among metabolic risk factors that are difficult to capture using traditional statistical approaches [6,9,10,11,12]. Importantly, these models can be developed using routinely available clinical and laboratory parameters, including anthropometric measures, glycemic indices, lipid profiles, and liver enzymes, thereby enabling non-invasive, low-cost, and scalable detection of hepatic steatosis.

Several classical supervised ML algorithms, including logistic regression, random forest, support vector machines, k-nearest neighbors, and boosting-based methods, have been applied to the prediction of fatty liver disease and ultrasound-based steatosis classification. For example, Weng et al. developed multiple ML models for fatty liver detection based on abdominal ultrasonography [13]. Similarly, Marques et al. evaluated ML classifiers for ultrasound-based hepatic steatosis classification [14]. A recent review by Mahzari et al. further summarized the application of these algorithms in fatty liver prediction, highlighting their established role in this clinical domain [15].

Despite growing interest, systematic comparisons of multiple ML models within leakage-free validation frameworks, together with clinically interpretable outputs, remain limited in the context of MASLD screening.

In this study, we developed and validated ML models to detect hepatic steatosis using exclusively routinely collected clinical and biochemical data. We systematically compared multiple algorithms, assessed model discrimination, and evaluated clinical interpretability and utility across actionable decision thresholds. To enhance transparency and facilitate clinical adoption, we further explored global feature importance and individual-level explanations.

Because the present study was based on structured tabular clinical data composed of routinely collected demographic, anthropometric, clinical, and biochemical variables, the evaluated algorithms were intentionally selected from established supervised learning approaches commonly used in clinical prediction tasks involving this type of input. This design enabled comparison across complementary modeling paradigms, including interpretable linear models and more flexible nonlinear and ensemble-based methods. Given the moderate sample size of the cohort, this modeling framework was considered methodologically appropriate, allowing robust internal validation while preserving clinical interpretability and applicability.

Accordingly, the aim of the present study was to develop an explainable, leakage-resistant, and clinically deployable predictive framework for ultrasound-detected steatosis within the MASLD spectrum using only routinely available clinical and biochemical parameters. This implementation-oriented approach was designed to facilitate integration into primary and metabolic care workflows.

## 2. Methods

### 2.1. Dataset and Ultrasonography Outcome Definition

This retrospective observational study used routinely collected clinical data from Ankara Etlik City Hospital, a tertiary referral center in Ankara, Türkiye. Adult patients (≥18 years) who underwent abdominal ultrasonography for any clinical indication between 1 May 2024 and 30 April 2025 were eligible for inclusion. The study protocol was reviewed and approved by the Scientific Research Evaluation and Ethics Committee of Ankara Etlik City Hospital, Ankara, Türkiye (Approval Date and ID: 22.10.2025, AEŞH-BADEK-2025-590).

Ultrasonography was performed using GE Healthcare ultrasound systems (GE Healthcare, Chicago, IL, USA) as part of standard clinical care and interpreted by experienced radiologists. Hepatic steatosis was assessed using established qualitative criteria based on increased hepatic echogenicity relative to the renal cortex and spleen, as documented in standardized radiology reports. The study outcome was ultrasound-detected hepatic steatosis, recorded as present or absent in the radiology report, and used as the supervised learning target representing hepatic steatosis within the MASLD spectrum.

Ultrasonography was deliberately selected as the reference imaging modality to define hepatic steatosis, as it remains the most widely accessible and routinely used first-line diagnostic tool in real-world clinical practice and population-based settings. Although its limitations in detecting mild steatosis, particularly near the 5% fat threshold, are well recognized, it represents the pragmatic standard for initial assessment within the MASLD spectrum. In the present study, ultrasonography was used exclusively to define the binary outcome variable (presence or absence of hepatic steatosis) for supervised ML model development. No ultrasound images were used as model inputs, and no manual or automated image-based feature extraction, quantitative attenuation measurements, radiomic analysis, or deep learning-based image processing were performed. All predictive variables were derived solely from routinely collected clinical, anthropometric, and biochemical data.

Patients were included if ultrasonography reports clearly documented the presence or absence of hepatic steatosis and if required predictor variables were available within 1 month of imaging. Individuals with missing ultrasonography outcomes were excluded. Additional exclusion criteria included significant alcohol consumption, known chronic viral hepatitis, other chronic liver diseases, or the use of hepatotoxic medications, when such information was available in the medical records, in order to align with the diagnostic framework of MASLD. The final dataset consisted of 644 patients, with 515 (80%) allocated to the training set and 129 (20%) to the test set using stratified random sampling.

The dataset used in the present study was derived from the institutional patient cohort contributed by our center to the previously published nationwide multicenter MASLD study by Kirik et al. [16]. In that multicenter investigation, the pooled dataset from several centers was used to estimate the national prevalence of MASLD and the risk of advanced fibrosis among individuals with cardiometabolic risk factors using conventional epidemiological analyses.

The present study represents a secondary, methodologically independent analysis of the institutional dataset from our center. Although a subset of patients overlaps with the cohort contributed to the previously published multicenter MASLD study, the research objective, analytical strategy, and outcomes of the present work are fundamentally different. Specifically, while the multicenter study focused on estimating epidemiological prevalence and fibrosis risk, the current study develops and evaluates ML models to predict ultrasound-detected hepatic steatosis using routinely available clinical and biochemical parameters.

Therefore, the present analysis addresses a distinct research question and should not be interpreted as a subdivision or reanalysis of the previously published study, and no analyses from that work were reused in the present manuscript.

In addition to the subset of patients that contributed to the multicenter dataset, additional eligible patients from the same institutional cohort who met identical inclusion criteria during the study period were incorporated into the present analysis. The inclusion of these additional cases was intended to increase the sample size and improve the statistical robustness of the ML models while maintaining identical variable definitions, outcome criteria, and data collection procedures. Accordingly, the dataset used in the present study represents an expanded institutional cohort rather than a structurally modified dataset.

### 2.2. Study Design and Modeling

The primary objective was to predict the presence of ultrasound-detected hepatic steatosis within the MASLD spectrum using supervised ML algorithms applied to routinely collected clinical, anthropometric, and biochemical variables. The target outcome was ultrasound-detected hepatic steatosis (present/absent). All analyses were performed in Python 3.11.5 using standard scientific libraries, including pandas (v2.1.1), numpy (v1.26.0), scikit-learn (v1.3.1), matplotlib (v3.8.0), and xgboost (v2.0.0) and SHapley Additive exPlanations (SHAP) (v0.44.0) for gradient boosting and model explainability, respectively.

### 2.3. Preprocessing and Feature Selection (Single Pipeline)

The ML models in the present study were trained using tabular clinical data derived from routinely collected electronic health records. The original dataset included 86 candidate variables per patient, encompassing demographic characteristics, anthropometric measurements, clinical comorbidities, medication use, routine laboratory parameters, and derived metabolic or cardiovascular indices. These variables are summarized in Table 1 and Table 2. Ultrasonography was used exclusively to define the binary outcome variable (presence or absence of hepatic steatosis) and was not used as an input feature in the ML models.

To prevent information leakage, all preprocessing and feature-selection procedures were implemented within a single scikit-learn pipeline and executed inside the cross-validation loop. To ensure methodological validity, preprocessing and feature-selection procedures were treated as integral components of the modeling pipeline rather than optional analytical steps, and therefore were embedded within the cross-validation framework to prevent information leakage and maintain unbiased performance estimation. Numeric variables were median-imputed and standardized to z-scores, whereas categorical variables were mode-imputed and one-hot encoded with unknown levels ignored at transform time.

Feature selection was performed using logistic regression with an Elastic Net penalty (SAGA solver), combining L1 regularization for sparsity and L2 regularization for stability under collinearity. Predictors with non-zero coefficients after regularization were considered selected. Because one-hot encoding generates multiple columns per original variable, variable-level importance was summarized as the L2 norm of all associated coefficients. Variables were ranked by this aggregated importance, and the top 20 variables were retained as the feature panel for subsequent modeling and evaluation, with rankings derived exclusively from the training partitions within each resampling iteration.

The evaluated algorithms were selected to represent complementary modeling strategies commonly used for structured tabular clinical data, allowing comparison between linear, nonlinear, and ensemble-based approaches within the same analytical pipeline.

### 2.4. Data Splitting and Resampling

To obtain an unbiased estimate of performance, patients were randomly allocated using stratified sampling to training (80%; n = 515) and test (20%; n = 129) sets, preserving the class distribution of ultrasound-detected hepatic steatosis vs. no ultrasound-detected hepatic steatosis. The same train–test partition was applied across all algorithms to ensure comparability. Within the training data, we used repeated stratified k-fold cross-validation with 5 folds and 5 repetitions (total 25 resamples; fixed random seed) to benchmark candidate models. At each fold, the full pipeline (imputation → encoding/standardization → Elastic-Net selection → classifier) was fitted on the training split and evaluated on the corresponding validation split.

To ensure fair and directly comparable evaluation across classifiers, the same stratified train–test split and identical random seed were used for all models. All cross-validation procedures were performed using fixed random states, and no classifier-specific resampling or data re-partitioning was applied. The held-out test set was reserved for independent evaluation, whereas the performance metrics reported in the Section 3 primarily reflect the repeated cross-validation estimates obtained from the training data, which provide a more stable assessment of model discrimination across resampling iterations.

### 2.5. Classifiers

The evaluated algorithms were selected to represent widely used classification approaches for tabular clinical datasets. This design allows for comparison between interpretable linear models, such as logistic regression, and more flexible nonlinear approaches, including ensemble and instance-based algorithms commonly applied in clinical prediction modeling. The evaluated classifiers comprised decision tree, AdaBoost [base estimator: decision tree; Stagewise Additive Modeling using a Multiclass Exponential loss function (SAMME)], random forest, XGBoost (eval_metric = logloss), gradient boosting, support vector machine with probability estimates enabled, k-nearest neighbors, multilayer perceptron (maximum iterations = 5000), Gaussian Naive Bayes, and logistic regression (maximum iterations = 2000). A unified benchmarking strategy was applied across all classifiers rather than extensive model-specific hyperparameter optimization. The classifiers were evaluated using standard scikit-learn and XGBoost implementations with largely predefined settings. Limited parameter adjustments were made only for technical or convergence-related reasons, including enabling probability estimates for support vector machines, setting the XGBoost evaluation metric to log loss, using the SAMME algorithm for AdaBoost, and increasing the maximum iteration limits for multilayer perceptron and logistic regression. No separate grid search, randomized search, Bayesian optimization, or model-specific hyperparameter tuning procedure was applied. This strategy was selected to ensure a consistent and comparable evaluation of different algorithm families within the same repeated stratified 5-fold cross-validation framework, while reducing the risk of overfitting in a moderate-sized retrospective cohort. For algorithms without native probability outputs, decision-function scores were used for receiver operating characteristic (ROC) analyses.

To provide methodological context, we evaluated a diverse set of supervised ML algorithms representing complementary modeling paradigms. Logistic regression was included as a transparent linear baseline with strong interpretability and well-calibrated probability estimates, but limited capacity to capture nonlinear relationships. Tree-based ensemble methods (decision tree, random forest, gradient boosting, and XGBoost) were selected for their ability to model nonlinear effects and higher-order interactions without explicit feature engineering; random forest emphasizes variance reduction through bagging, whereas boosting-based methods prioritize bias reduction through sequential error correction, at the cost of increased complexity and reduced interpretability.

Support vector machines were included for their effectiveness in high-dimensional spaces and robustness to overfitting, although probability calibration may be less reliable. k-nearest neighbors was evaluated as a nonparametric, instance-based learner sensitive to local structure but prone to performance degradation in high-dimensional settings. Gaussian naïve Bayes provided a probabilistic baseline with strong bias assumptions and computational efficiency, while the multilayer perceptron represented a shallow neural-network approach capable of modeling nonlinearities but sensitive to sample size and hyperparameter choices. Collectively, this model set enabled a balanced comparison between interpretability, flexibility, and predictive performance.

### 2.6. Evaluation Metrics and Interpretability

Predictive performance was summarized using accuracy, sensitivity (recall), specificity, positive and negative predictive values (PPVs/NPVs), F1 score, Youden’s J index, balanced accuracy, and area under the receiver operating characteristic curve (AUROC). Receiver-operating-characteristic curves were generated to visualize diagnostic discrimination. For each algorithm, results are reported as mean ± standard deviation across the 25 resamples to enable robust, distribution-aware comparisons. For interpretability, we additionally fitted a logistic-regression model (solver = liblinear) on standardized predictors and applied SHAP to quantify feature contributions, highlighting clinical variables with consistently high impact on ultrasound-detected hepatic steatosis.

In addition to commonly reported performance metrics such as accuracy, precision, recall, and F1 score, we report Youden’s J index to provide a threshold-independent summary of diagnostic effectiveness. Youden’s J (sensitivity + specificity − 1) quantifies the maximum achievable separation between true-positive and false-positive rates and is widely used in diagnostic and screening settings where balanced performance across classes is clinically important. In the context of MASLD screening, where both missed cases (false negatives) and unnecessary follow-up investigations (false positives) carry clinical and economic consequences, Youden’s J offers complementary information by emphasizing the sensitivity–specificity trade-off rather than overall correctness alone.

In addition to overall accuracy, we report balanced accuracy, defined as the average of sensitivity and specificity. In binary classification, balanced accuracy is equivalent to macro-averaged accuracy, as it weights both classes equally. Although the dataset is approximately balanced, balanced accuracy provides a class-symmetric assessment that is independent of class prevalence and facilitates comparison with diagnostic metrics such as sensitivity, specificity, and Youden’s J index. For ML models, classification-based performance metrics (sensitivity, specificity, accuracy, balanced accuracy, and Youden’s J) were computed using a fixed, pre-specified probability threshold of 0.5, applied consistently across all cross-validation resamples and the test set.

### 2.7. Comparative Evaluation of Simple Steatosis Scores and ML Models

To contextualize the clinical performance of the proposed ML models, we evaluated two widely used rule-based steatosis indices, HSI and FLI, using their originally proposed diagnostic thresholds. Importantly, these indices were applied as intended, without recalibration, refitting, or threshold optimization [17,18].

For HSI, values < 30 were considered to rule out hepatic steatosis, values ≥ 36 to rule in steatosis, and values between 30 and 35.9 were classified as indeterminate and excluded from performance analyses. For FLI, values < 30 were considered to rule out steatosis, values ≥ 60 to rule in steatosis, and values between 30 and 59.9 were considered indeterminate and excluded. Diagnostic performance metrics for HSI and FLI were calculated only among classifiable patients, in accordance with their original design [18]. Sensitivity, specificity, accuracy, and AUROC were calculated using ultrasound-detected hepatic steatosis as the reference standard. For rule-based indices, AUROC was computed using the binary rule-based outputs after exclusion of indeterminate cases. In contrast, AUROC for ML models was calculated using continuous predicted probabilities across the entire cohort. This approach avoids implicit recalibration of established indices while allowing a fair, clinically meaningful comparison of discrimination and population coverage.

## 3. Results

### 3.1. Baseline Characteristics

Baseline demographic, anthropometric, clinical, and biochemical characteristics of participants according to hepatic steatosis status are summarized in Table 1. Among 644 individuals, 322 (50%) had ultrasound-detected hepatic steatosis and 322 (50%) had normal liver echogenicity.

Participants with hepatic steatosis were younger on average (59.8 ± 16.0 vs. 62.4 ± 19.1 years, *p* = 0.004). Sex distribution and educational status were comparable between groups (*p* > 0.05). Anthropometric measures revealed substantial differences: individuals with hepatic steatosis exhibited greater body weight (79.8 ± 18.2 vs. 70.4 ± 14.6 kg, *p* < 0.001), higher body mass index (BMI) (29.0 ± 6.6 vs. 26.2 ± 5.8 kg/m^2^, *p* < 0.001), and larger waist circumference (95.7 ± 18.2 vs. 87.7 ± 17.2 cm, *p* < 0.001). They were also slightly taller (165.9 ± 10.0 vs. 164.2 ± 10.2 cm, *p* = 0.043). Regular weekly exercise was significantly less frequent in the hepatic steatosis group (13.7% vs. 22.1%, *p* = 0.005).

Hemodynamic parameters showed modest but significant elevations in the hepatic steatosis cohort: systolic blood pressure (126.6 ± 17.3 vs. 124.3 ± 16.1 mmHg, *p* = 0.026), diastolic blood pressure (75.2 ± 11.1 vs. 72.6 ± 9.7 mmHg, *p* < 0.001), and heart rate (82.7 ± 13.2 vs. 80.4 ± 12.9 bpm, *p* = 0.031).

Regarding comorbidities, diabetes mellitus (51.2% vs. 36.0%, *p* < 0.001) and dyslipidemia (32.0% vs. 18.3%, *p* < 0.001) were significantly more prevalent in participants with ultrasound-detected hepatic steatosis, while hypertension and cardiovascular diseases did not differ significantly. Obstructive sleep apnea was more common among hepatic steatosis participants (1.6% vs. 0%, *p* = 0.025). Medication use paralleled disease prevalence: metformin (25.8% vs. 12.4%, *p* < 0.001), sodium–glucose cotransporter-2 inhibitors (SGLT2-i) (14.0% vs. 8.1%, *p* = 0.017), and statins (20.2% vs. 12.1%, *p* = 0.005) were all used more frequently in participants with ultrasound-detected hepatic steatosis.

Hematologic evaluation showed slightly higher hemoglobin levels (11.8 ± 2.6 vs. 11.1 ± 2.6 g/dL, *p* < 0.001) and modestly lower lymphocyte counts (*p* = 0.004) in the hepatic steatosis group, whereas other cell counts were comparable.

Biochemically, participants with ultrasound-detected hepatic steatosis had a more adverse metabolic profile characterized by higher fasting glucose (144.5 ± 81.2 vs. 126.1 ± 63.3 mg/dL, *p* < 0.001), glycated hemoglobin (HbA1c) (7.11 ± 2.78 vs. 6.51 ± 2.34%, *p* < 0.001), and triglycerides (170.9 ± 116.7 vs. 134.4 ± 153.6 mg/dL, *p* < 0.001). Total cholesterol was slightly higher (*p* = 0.039), while low-density lipoprotein (LDL) and high-density lipoprotein (HDL) cholesterol did not differ significantly.

Liver enzymes did not show a uniform pattern of elevation in the hepatic steatosis group. AST and GGT were numerically higher but did not reach statistical significance, whereas ALT did not demonstrate a higher mean value in participants with hepatic steatosis. Albumin was higher in participants with ultrasound-detected hepatic steatosis (37.7 ± 4.6 vs. 36.0 ± 5.1 g/L, *p* < 0.001), while bilirubin fractions, blood urea nitrogen (BUN), creatinine (Cr), estimated glomerular filtration rate (eGFR), and thyroid indices were comparable (*p* > 0.05).

Overall, participants with hepatic steatosis exhibited greater adiposity, insulin resistance, and metabolic derangements, consistent with metabolic dysfunction-related patterns observed within the MASLD spectrum (Table 1). Derived anthropometric, metabolic, cardiovascular, hepatic, hematologic, and renal indices are summarized in Table 2.

Participants with ultrasound-detected hepatic steatosis demonstrated consistently higher values across multiple body composition indices, including waist-to-height ratio, body fat percentage, ponderal index, conicity index, relative fat mass, and visceral adiposity index (all *p* < 0.01), reflecting excess central and visceral adiposity, key drivers of hepatic fat accumulation.

Metabolic indices integrating glycemic status and lipid metabolism were markedly elevated in the ultrasound-detected hepatic steatosis group. The triglyceride–glucose index (TyG), TyG-BMI, TyG–waist circumference (WC), TyG–triglyceride–glucose index adjusted for waist-to-height ratio (WHtR), lipid accumulation product (LAP), and atherogenic index of plasma (AIP) were all significantly higher (all *p* < 0.001), indicating greater insulin resistance and dyslipidemia among individuals with steatosis. These composite indices capture nonlinear metabolic interactions that are not fully represented by isolated glucose or lipid measurements.

Cardiovascular risk indices, including Castelli I and II ratios, non-HDL cholesterol, remnant cholesterol, and rate pressure product, were also significantly increased in the hepatic steatosis group, underscoring the close association between hepatic steatosis and adverse cardiometabolic risk profiles. In contrast, pulse pressure did not differ between groups.

Regarding liver-related indices, the HSI was substantially higher in participants with ultrasound-detected hepatic steatosis (*p* < 0.001), supporting internal consistency with the ultrasound-based outcome definition. While fibrosis-oriented scores such as aspartate aminotransferase (AST)-to-platelet ratio index (APRI), fibrosis-4 index (FIB-4), and nonalcoholic fatty liver disease (NAFLD) fibrosis score did not differ significantly, this likely reflects the predominance of early-stage steatosis rather than advanced fibrotic disease in the cohort. Albumin–bilirubin score and hemoglobin–albumin–lymphocyte–platelet score (HALP) score differed modestly but significantly, suggesting subtle alterations in hepatic synthetic function and systemic inflammatory–nutritional balance.

Finally, selected renal and metabolic ratios, including uric acid-to-HDL ratio, were higher in the hepatic steatosis group, consistent with emerging evidence linking hyperuricemia and renal–metabolic interactions to steatotic liver disease. Overall, Table 2 demonstrates that hepatic steatosis is associated with a broad constellation of adverse adiposity-related, metabolic, cardiovascular, and hepatic indices, many of which later emerged as key predictors in the ML models.

### 3.2. Model Development and Evaluation

**Figure 1:**
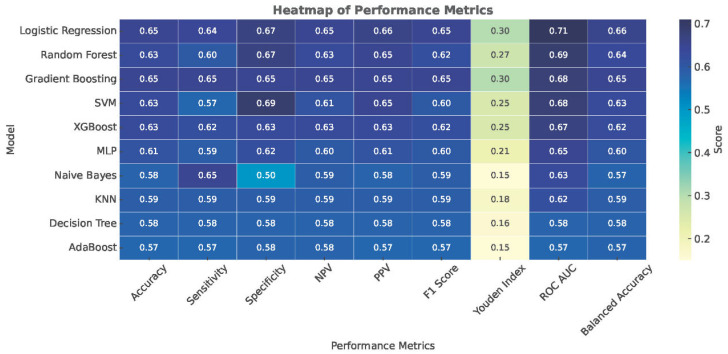
Heatmap visualization of the performance of machine learning classifiers for predicting ultrasound-detected hepatic steatosis. Performance metrics including accuracy, sensitivity, specificity, negative predictive value (NPV), positive predictive value (PPV), F1-score, Youden index, ROC AUC, and balanced accuracy are shown for each model. Color intensity reflects the magnitude of the performance metrics. Logistic Regression, Gradient Boosting, and Random Forest demonstrated the highest overall predictive performance, with ROC AUC values ranging from 0.67 to 0.71.

We trained and evaluated multiple supervised classifiers to discriminate ultrasound-detected hepatic steatosis from its absence using routinely collected clinical, anthropometric, and biochemical variables. Model performance was assessed using repeated stratified cross-validation and summarized by accuracy, sensitivity, specificity, predictive values, F1 score, Youden’s J, balanced accuracy, and AUROC (Table 3, Figure 1).

To examine the contribution of preprocessing and feature selection, we compared model performance using the full original feature set and the Elastic Net-selected 20-feature panel within the same cross-validation framework. Across models, performance with the reduced feature panel was comparable to, and in some cases modestly improved over, the full feature set, while showing lower variance across cross-validation folds. These findings indicate that the primary performance gains were driven by the removal of redundant and collinear variables, resulting in more stable and interpretable models without loss of discrimination.

### 3.3. Elastic Net-Based Variable Selection

The Elastic Net procedure converged and produced a sparse solution, shrinking many coefficients to zero while retaining a compact set of informative predictors. To avoid information leakage, preprocessing and selection were implemented inside a single scikit-learn pipeline during cross-validation. Numeric variables were median-imputed and standardized (z-score); categorical variables were mode-imputed and one-hot encoded with unknown levels ignored at transform time. Predictors (or one-hot levels) with non-zero coefficients after regularization were considered selected. Because one-hot encoding yields multiple columns per original variable, variable-level importance was summarized as the L2 norm of all associated coefficients.

Based on aggregated variable-level importance, the top 20 variables associated with ultrasound-detected hepatic steatosis were (in descending order of aggregated importance): Weight (kg), Ponderal index, FIB-4, BUN/Cr ratio, height (cm), APRI, Castelli II, VAI (Visceral adiposity index), LDL cholesterol (mg/dL), TyG-WC, uric acid (UA)/Cr Ratio, BUN (mg/dL), NAFLD fibrosis score, albumin (g/L), AST (IU/L), TyG-BMI, ALT (IU/L), weekly exercise history, Cr (mg/dL), and a body shape index (ABSI). These variables appear in descending aggregated importance in Supplementary Figure S1, while encoded-level effects (e.g., per one-hot level) are summarized in Supplementary Figure S2. In the logistic framework, positive coefficients indicate higher log-odds of hepatic steatosis and negative coefficients indicate lower log-odds. This feature-selection step establishes a fixed 20-variable panel for all subsequent model development and reporting.

### 3.4. Accuracy

Overall accuracy was moderate. The highest mean accuracies were observed for Logistic Regression and Gradient Boosting (each 0.65 ± 0.03), followed by Random Forest and Support Vector Machine (SVM)/XGBoost (≈0.63 ± 0.03–0.04). Lower-tier models achieved ≤0.61 on average [Multilayer Perceptron (MLP) 0.61 ± 0.04; k-NN 0.59 ± 0.03; Decision Tree 0.58 ± 0.04; Naïve Bayes 0.58 ± 0.06; AdaBoost 0.57 ± 0.0]. Thus, Logistic Regression and Gradient Boosting were the most accurate learners.

### 3.5. F1 Score

F1 scores mirrored accuracy: Logistic Regression and Gradient Boosting led with 0.65 ± 0.04, while Random Forest/XGBoost were slightly lower (≈0.62 ± 0.03–0.04). SVM/MLP were around 0.60 ± 0.04; k-NN 0.59 ± 0.04, Decision Tree 0.58 ± 0.05, Naïve Bayes 0.59 ± 0.11, and AdaBoost 0.57 ± 0.05. Hence, Logistic Regression and Gradient Boosting achieved the best precision–recall balance.

### 3.6. Sensitivity

Mean sensitivity was highest for Gradient Boosting (0.65 ± 0.06) and Logistic Regression (0.64 ± 0.06). Naïve Bayes reached a similar mean (0.65 ± 0.20) but with very wide dispersion, indicating instability. SVM traded sensitivity (0.57 ± 0.04) for higher specificity (see below).

### 3.7. Specificity

Specificity peaked with SVM (0.69 ± 0.06), followed by Random Forest (0.67 ± 0.05) and Logistic Regression (0.67 ± 0.06). Gradient Boosting yielded 0.65 ± 0.06. Thus, the most conservative false-positive control was achieved by SVM.

### 3.8. Youden’s J

Youden’s J was highest for Logistic Regression and Gradient Boosting (each 0.30 ± 0.07), then Random Forest (0.27 ± 0.07) and SVM/XGBoost (≈0.25 ± 0.06–0.07). Lower-tier models were ≤0.21 (e.g., MLP 0.21 ± 0.07; k-NN 0.18 ± 0.07; Decision Tree 0.16 ± 0.08; Naïve Bayes 0.15 ± 0.12; AdaBoost 0.15 ± 0.08). Accordingly, Logistic Regression and Gradient Boosting maximized the sensitivity–specificity composite.

### 3.9. AUROC

Mean AUROC was highest for Logistic Regression (0.71 ± 0.04), followed by Random Forest (0.69 ± 0.04), Gradient Boosting (0.68 ± 0.04), SVM (0.68 ± 0.04), and XGBoost (0.67 ± 0.03). Remaining models were ≤0.65 (MLP 0.65 ± 0.03, Naïve Bayes 0.63 ± 0.06, k-NN 0.62 ± 0.04, Decision Tree 0.58 ± 0.04, AdaBoost 0.57 ± 0.04).

### 3.10. Balanced Accuracy

Balanced accuracy corroborated the above: Logistic Regression (0.66 ± 0.06) and Gradient Boosting (0.65 ± 0.06) led, with Random Forest (0.64 ± 0.04), SVM (0.63 ± 0.05), and XGBoost (0.62 ± 0.06) close behind; other models were ≤0.60.

### 3.11. ROC Visualization

**Figure 2:**
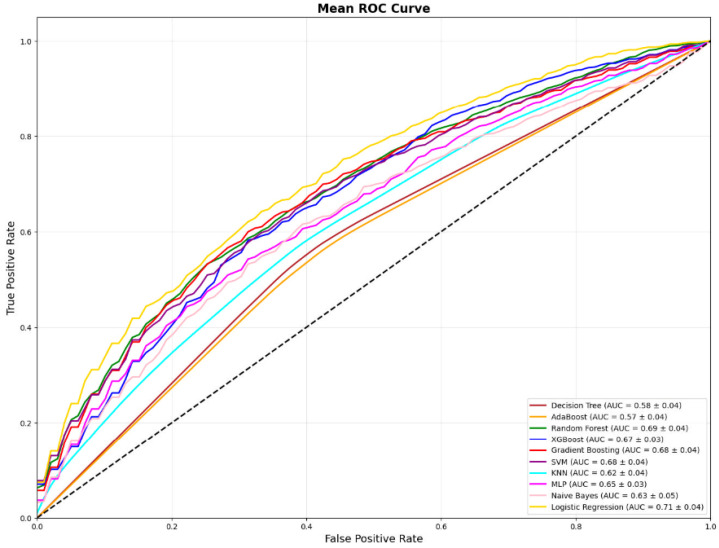
Mean receiver operating characteristic (ROC) curves of machine learning classifiers for predicting ultrasound-detected hepatic steatosis. Curves represent the mean ROC performance across 25 resampling iterations. The dashed diagonal line indicates random classification. Logistic Regression demonstrated the highest discriminative performance (AUC = 0.71), followed by Random Forest (AUC = 0.69) and Gradient Boosting (AUC = 0.68).

Mean ROC curves (Figure 2) showed a reproducible lift above chance for the top-tier models, consistent with their AUROC ranking in Table 3. Upper-envelope curves corresponded to Logistic Regression, Random Forest, Gradient Boosting, SVM, and XGBoost (Figure 2; Table 3).

### 3.12. PPV/NPV

PPV/NPV reflected each model’s sensitivity–specificity balance. For example, Logistic Regression achieved PPV 0.66 ± 0.04 and NPV 0.65 ± 0.03, while SVM showed PPV 0.65 ± 0.05 with lower NPV (0.61 ± 0.03) owing to its specificity-oriented profile. Random Forest and Gradient Boosting remained in the mid-0.60s for both indices. Hence, Logistic Regression and Gradient Boosting offered the most balanced predictive value profiles among top performers (Table 3).

### 3.13. SHAP Summaries and Directionality

**Figure 3:**
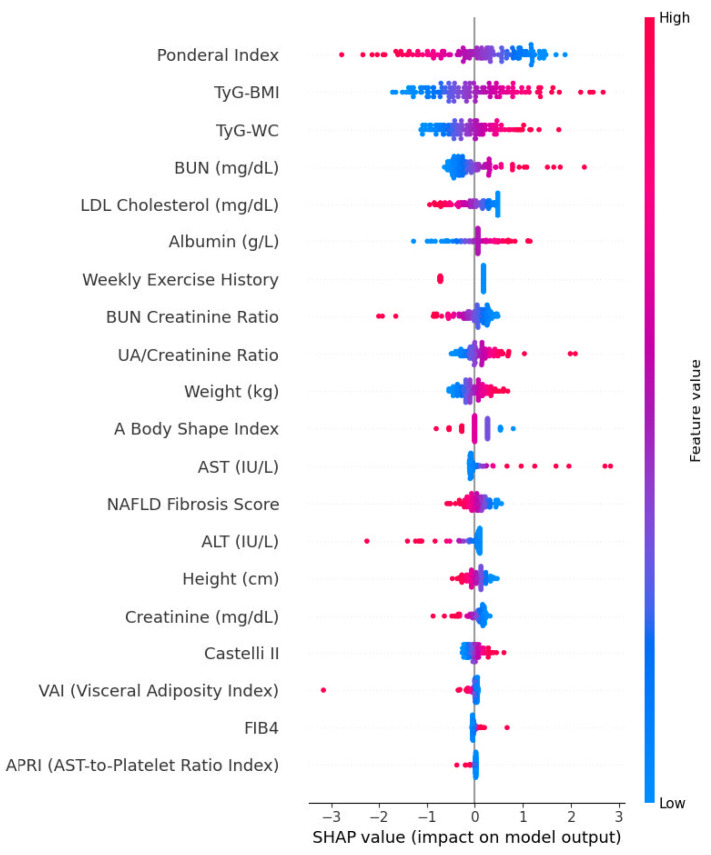
SHAP summary plot illustrating the contributions of individual variables to the logistic regression model for predicting ultrasound-detected hepatic steatosis. Each dot represents the SHAP value of a single observation; color indicates the magnitude of the feature values (red = higher values, blue = lower values). Positive SHAP values increase the predicted probability of ultrasound-detected hepatic steatosis. Anthropometric and metabolic features, including ponderal index and TyG-derived indices, as well as markers related to hepatic and renal function, showed the largest contributions to model predictions.

To elucidate model behavior, we computed SHAP summary plots from a logistic-regression explainer fitted on standardized predictors (Figure 3). The global importance profile showed a right-skewed distribution, indicating that a compact subset of the selected variables accounts for most of the predictive signal. The direction of effects was clinically coherent: features with higher SHAP magnitudes systematically increased (positive SHAP) or decreased (negative SHAP) the predicted probability of ultrasound-detected hepatic steatosis in line with their regularized coefficients. Local (beeswarm) patterns revealed heterogeneous but directionally stable effects across individuals, with no single feature exerting idiosyncratic influence limited to a narrow subgroup. Collectively, the Elastic Net ranking and SHAP attributions were concordant, reinforcing that the model relies on interpretable, routinely available parameters, and supporting the plausibility and robustness of observed discrimination. Although Elastic Net-based feature selection identified a clinically coherent predictor set, we did not perform a formal fold-wise feature stability analysis based on selection frequencies across all cross-validation resamples. Therefore, the reported feature panel should be interpreted as a parsimonious and clinically interpretable predictor set rather than a definitive ranking of biomarker importance. Given the presence of correlated anthropometric, metabolic, hepatic, and lipid-derived indices, some variability in selected predictors across resampling iterations would be expected. Future studies using larger external cohorts should further evaluate feature stability through repeated selection-frequency analyses or bootstrap-based stability selection approaches.

### 3.14. Comparison Between ML Models and Simple Clinic Scores for Ultrasound-Detected Hepatic Steatosis

When applied according to their original diagnostic rules, both HSI and FLI demonstrated modest discriminative ability and left a substantial proportion of patients in an indeterminate category (Table 4). Using rule-based classification, HSI was applicable to 71.3% of the cohort, excluding 28.7% as indeterminate, and achieved high sensitivity (0.84) but limited specificity (0.42), reflecting its primary role as a rule-out tool. FLI was applicable to 76.6% of patients, excluding 23.4% as indeterminate, and showed a more balanced but still moderate performance (sensitivity 0.71, specificity 0.55). The AUROC values for rule-based HSI and FLI were 0.63 and 0.63, respectively. In contrast, the best-performing ML models, logistic regression and gradient boosting, provided continuous risk estimates for 100% of patients, without an indeterminate zone. These models demonstrated higher overall discrimination, with AUROC values of approximately 0.71 for logistic regression and 0.68 for gradient boosting, alongside balanced accuracy values of approximately 0.65–0.66. Unlike rule-based indices, ML models maintained stable performance across repeated cross-validation and allowed flexible threshold selection depending on clinical context. Collectively, these findings indicate that while HSI and FLI retain utility as simple screening tools, ML models offer improved discrimination, complete cohort coverage, and greater flexibility for individualized risk stratification.

## 4. Discussion

Metabolic dysfunction-associated steatotic liver disease has emerged as the most prevalent chronic liver condition worldwide, paralleling the global rise in obesity, type 2 diabetes, and metabolic syndrome. Early identification of individuals at risk is critical, yet conventional diagnostic modalities such as ultrasonography, CAP, or MRI-PDFF remain costly, operator dependent, and impractical for population-level screening [19,20]. In this study, we developed and validated ML models leveraging routine clinical and biochemical parameters to predict ultrasound-detected hepatic steatosis. Among the evaluated algorithms, logistic regression and gradient boosting achieved the highest performance, with an AUROC of 0.70 and a balanced accuracy of 0.65, demonstrating modestly improved discrimination compared with rule-based indices such as FLI and HSI in our cohort, while avoiding indeterminate classifications and ensuring complete population coverage [7,9,17,21]. An important methodological consideration when comparing ML models with established rule-based indices is population coverage. HSI and FLI are designed with rule-in and rule-out thresholds and therefore include an intermediate indeterminate range. Accordingly, their diagnostic performance is evaluated only among individuals classified outside this gray zone, whereas ML models generate probability-based predictions for all patients. This difference may influence the interpretability of direct metric-based comparisons, because exclusion of indeterminate cases alters the evaluable population. Therefore, comparisons between ML models and HSI/FLI should be interpreted not only in terms of discrimination metrics, but also in the context of clinical applicability and cohort coverage across the target population. These findings suggest that the moderate performance observed across models may be improved by incorporating larger and more diverse datasets, integrating imaging-derived or longitudinal features, and performing external validation in independent cohorts. The present model is designed to predict ultrasound-detected hepatic steatosis, which represents an early and central component of the MASLD spectrum, rather than establishing a comprehensive diagnosis of MASLD that requires broader clinical and metabolic evaluation. Although several cardiometabolic and cardiovascular indices were included among the candidate predictors, the present framework should not be interpreted as a model for predicting MASLD progression, fibrosis development, cardiovascular disease onset, or longitudinal cardiovascular risk trajectories. The study was cross-sectional in design, and the supervised learning target was limited to the binary classification of current ultrasound-detected hepatic steatosis. Therefore, lipid-, insulin resistance-, and cardiovascular-related markers included in the model should be interpreted as cross-sectional correlates that improve classification of current liver status rather than as predictors of future cardiovascular events or temporal disease progression. Longitudinal cohorts with time-to-event outcomes will be required to determine whether similar ML frameworks can predict longitudinal liver- and cardiovascular-related outcomes.

The comparable performance of Logistic Regression and Gradient Boosting suggests that a substantial proportion of the predictive signal may be captured by additive effects of routinely measured clinical variables, although more complex nonlinear interactions cannot be excluded. The modest yet consistent AUROC observed in the present study is broadly consistent with recent ML approaches for MASLD or steatosis prediction based on routinely available clinical and laboratory data, which similarly demonstrate moderate to good discrimination without reliance on imaging-derived features [22]. For example, Cubillos et al. showed that a novel deep learning (DL) approach, which converts tabular clinical data into image-like representations, outperformed traditional ML models and the HSI for predicting steatotic liver disease (SLD). Using data from 2999 patients, their best DL model achieved high diagnostic accuracy (AUC = 0.87, sensitivity = 0.95, specificity = 0.64), demonstrating the superior predictive capability of DL-based methods for non-invasive SLD detection [23]. Lim et al. developed and validated ML-based survival models to predict the time to onset of MASLD in individuals without baseline disease. Using data from over 25,000 Korean participants for model development and 16,000 Chinese participants for independent validation, they trained random survival forest and extra survival tree models based on routine clinical and laboratory variables. Both models demonstrated strong predictive performance, with c-indices around 0.75 in the external cohort. The study showed that ML survival models can accurately estimate individualized risk and timing of MASLD onset, supporting personalized prediction and tailored follow-up strategies in clinical practice [24]. Our findings extend these observations by demonstrating model reproducibility across multiple algorithms and providing rigorous cross-validation estimates with embedded feature selection, thereby mitigating overfitting and information leakage.

Elastic Net regularization identified a concise set of 20 predictors reflecting both metabolic and hepatic injury pathways. Anthropometric indices (weight, Ponderal index, height, a body shape index) captured central and overall adiposity, consistent with the pivotal role of excess fat mass in hepatic lipid deposition [25,26]. Derived composite indices such as VAI, TyG-BMI, and TyG-WC integrate dyslipidemia and insulin resistance and are increasingly recognized as robust surrogates for visceral adiposity and hepatic steatosis [27,28].

Liver injury markers including ALT, AST, and fibrosis surrogates (FIB-4, APRI, NAFLD Fibrosis Score) contributed substantially, underscoring the continuum between metabolic steatosis and early fibrotic remodeling [29,30,31]. Renal and nitrogenous markers (BUN, Cr, BUN/Cr ratio, UA/Cr ratio) emerged as informative correlates, an observation supported by emerging data linking hyperuricemia and renal–hepatic axis dysfunction to steatosis and metabolic syndrome progression [32,33]. The inclusion of Castelli II index (LDL/HDL ratio) and albumin further reflects the interplay between lipid transport, synthetic function, and systemic inflammation [34,35].

Our model’s discrimination is broadly consistent with prior ML frameworks for the prediction of hepatic steatosis within the MASLD spectrum using routine data. Xiao et al. developed and validated five ML models, logistic regression, random forest, XGBoost, gradient boosting, and SVM, to predict MASLD using clinical and biochemical variables. In a cohort of 578 ultrasound-evaluated participants and an external MRI-based validation set (n = 131), key predictors included VAI, abdominal circumference, BMI, ALT, ALT/AST ratio, age, HDL-C, and triglycerides. Among the models, XGBoost achieved the highest predictive accuracy (AUC = 0.94 after tuning), outperforming others. The authors concluded that XGBoost offers a reliable, noninvasive tool for early identification of high-risk NAFLD patients in clinical settings [36]. Verschuren et al. developed a mechanism-based, non-invasive biomarker panel to detect fibrosis in MASLD [37]. Using a translational approach that integrated findings from a diet-induced MASLD mouse model with human liver transcriptomics and serum proteomics, they identified three key biomarkers: Insulin-Like Growth Factor Binding Protein 7 (IGFBP7), Scavenger Receptor Cysteine-Rich Type 5 Domain-Containing Protein (SSc5D), and Semaphorin 4D (Sema4D). When modeled using light gradient boosting machine (LightGBM), this panel accurately predicted fibrosis stages (AUCs = 0.82 for F0/F1, 0.89 for F2, 0.87 for F3/F4), outperforming established markers such as FIB-4, APRI, and FibroScan. The findings demonstrate that this three-protein blood-based panel can reliably identify both mild and advanced MASLD fibrosis, offering a promising non-invasive diagnostic tool. Although emerging serum, genetic, and omics-based biomarkers have demonstrated considerable potential for improving the detection, staging, and prognostic stratification of MASLD, their current clinical utility remains limited. Many of these biomarkers are associated with high costs, lack standardized analytical assays or universally accepted cut-off values, and are not routinely available in primary care or general clinical settings. In contrast, the present study deliberately prioritized real-world clinical applicability by exclusively utilizing routinely collected clinical, anthropometric, and biochemical parameters that are already embedded in standard care pathways. This design choice represents a pragmatic and implementation-oriented strategy rather than a restriction in conceptual scope. Importantly, future incorporation of well-validated biomarkers into this framework may further enhance predictive performance, particularly with respect to disease staging and progression, while preserving the scalability, accessibility, and clinical feasibility of the proposed models. This level of discrimination should therefore be interpreted in the context of the intentionally pragmatic design of the present study, which relied exclusively on routinely available clinical and biochemical parameters and applied leakage-free validation procedures, both of which tend to yield more conservative but clinically realistic performance estimates [38]. Importantly, the strict cross-validation strategy and embedded preprocessing design enhance model robustness and reduce optimistic bias, a limitation frequently observed in earlier single-split studies [26]. In the present study, model performance was primarily summarized as mean ± standard deviation across the 25 cross-validation resamples in order to reflect variability across repeated training–validation partitions. Because repeated cross-validation folds are not fully independent observations, these summaries should be interpreted as descriptive indicators of model stability rather than as formal measures of sampling uncertainty. For completeness, 95% confidence intervals were also reported in Table 3; however, these should be interpreted cautiously in the context of repeated resampling, where dependence between folds may limit the strict inferential meaning of conventional interval estimates.

In this context, reporting mean performance together with the standard deviation across resamples is widely used to characterize model robustness and variability across resampling iterations. Within this framework, the combined presentation of mean ± standard deviation and confidence intervals provides complementary descriptive information, while the overall interpretation should remain focused on consistency of performance across resampling iterations rather than on formal hypothesis-driven inference.

Interpretability remains central to the translation of ML tools into clinical workflows. SHAP analysis confirmed that model decisions were biologically plausible and aligned with established MASLD pathophysiology [39]. Higher weight, VAI, TyG-BMI, and FIB-4 consistently increased predicted risk, while higher albumin and lower Cr were protective. The concordance between Elastic Net coefficients and SHAP attributions reinforces model transparency and trustworthiness. Such interpretability facilitates clinician acceptance and supports integration into electronic health record (EHR)-based decision support systems, allowing automated, low-cost pre-screening for individuals warranting confirmatory imaging or lifestyle intervention.

From a public health perspective, scalable ML models based solely on routine clinical and biochemical data offer a pragmatic path toward early identification of hepatic steatosis within the MASLD spectrum in primary care. They can augment conventional scores by incorporating complex, multidimensional patterns without requiring novel biomarkers or imaging. Future work should focus on external validation across diverse ethnic groups and healthcare systems, incorporation of longitudinal data to predict disease progression such as fibrosis development, and integration of genetic and metabolomic predictors for enhanced precision [13,40].

While multiple prior studies have applied ML models with explainability to ultrasound-detected hepatic steatosis within the MASLD spectrum, the novelty of the present work lies not in introducing a new algorithm but in providing a methodologically rigorous and clinically deployable modeling framework for ultrasound-detected steatosis within the MASLD spectrum. In contrast to many earlier reports that rely on single train-test splits or post hoc feature interpretation, our approach integrates leakage-free preprocessing, embedded Elastic Net-based feature selection, repeated stratified cross-validation, and multi-model benchmarking under a unified pipeline. This design minimizes optimistic bias and enables fair comparison across distinct model families, prioritizing methodological rigor, interpretability, and real-world usability. Importantly, the primary objective of this study was not algorithmic innovation, but the construction of robust, transparent, and scalable tools that can operate on routinely collected clinical and biochemical data in routine hepatology and metabolic practice. Although deep learning architectures such as convolutional neural networks or transformer-based models may achieve higher classification performance when applied to raw imaging or high-dimensional data, such approaches typically require large annotated datasets, specialized computational infrastructure, and often provide limited interpretability. In contrast, the present study deliberately focused on routinely collected clinical and biochemical parameters to develop a transparent and clinically applicable screening framework, offering scalability, accessibility, and immediate applicability in primary care and metabolic clinics where advanced imaging data or deep learning infrastructure are not consistently available.

Furthermore, the study emphasizes clinical realism and interpretability, leveraging exclusively routinely collected variables and demonstrating that transparent models such as logistic regression and gradient boosting can achieve competitive performance when developed under robust validation protocols. The concordance between Elastic Net rankings and SHAP attributions reinforces biological plausibility and supports clinical trust, an aspect often underexplored in prior work focused primarily on performance metrics. Although direct benchmarking against publicly available datasets would be informative, heterogeneity in outcome definitions, population characteristics, and available predictors across MASLD datasets limits the validity of direct comparisons. Future studies should focus on harmonized external validation and benchmarking across multiple open datasets to further evaluate generalizability and comparative performance.

In this study, we compared interpretable ML models with established rule-based steatosis indices under conditions that preserve the original intent of each method. Rather than recalibrating or optimizing FLI and HSI within the study cohort, we applied their predefined rule-in and rule-out thresholds, thereby reflecting real-world clinical use. Our findings demonstrate that HSI and FLI, when used as designed, achieve modest discrimination (AUROC = 0.63) and leave approximately one quarter of patients in an indeterminate category. This limitation is inherent to their rule-based structure and reflects a deliberate trade-off between diagnostic certainty and coverage. HSI prioritizes sensitivity and exclusion of disease, whereas FLI offers a more balanced profile but still lacks comprehensive applicability. By contrast, the proposed ML models provide continuous, probabilistic risk estimates for all individuals, avoiding indeterminate classifications altogether. Although the absolute improvement in AUROC is modest, this gain becomes clinically meaningful when combined with full population coverage, balanced sensitivity–specificity trade-offs, and transparent model behavior. This advantage is particularly relevant in screening settings, where probabilistic risk estimation may offer practical benefits over rule-based scores that leave a substantial proportion of individuals unclassified. The interpretability of the ML models, supported by Elastic Net feature selection and SHAP analyses, further demonstrates that predictions are driven by biologically plausible metabolic and hepatic injury pathways, addressing a common barrier to clinical adoption of ML-based tools. Taken together, these results suggest that ML models should be viewed not as replacements for simple indices, but as complementary decision-support tools that can augment existing screening strategies. In settings where indeterminate results from rule-based scores necessitate further testing or imaging, ML-based risk stratification may help guide prioritization, reduce uncertainty, and support screening for ultrasound-detected hepatic steatosis within routine clinical workflows. It should also be noted that rule-based indices such as HSI and FLI inherently include indeterminate zones; consequently, their performance metrics are calculated only among classifiable individuals. In contrast, ML models generate continuous risk estimates for all patients. Accordingly, the comparison should be interpreted primarily in terms of clinical applicability and population coverage rather than as a purely head-to-head statistical equivalence.

Consistent with this observation, the comparative analyses suggest that the primary performance gains were not attributable to a specific classifier alone, but rather to the combined effect of leakage-free preprocessing and targeted feature reduction. In particular, the Elastic Net-derived feature panel achieved similar discrimination to the full feature set while improving model stability and interpretability.

In the present study, preprocessing and feature selection were integrated within a single leakage-free modeling pipeline. All imputation, scaling, encoding, and Elastic Net-based selection steps were embedded within the cross-validation loop, ensuring that performance estimates reflected unbiased model development procedures and the combined effects of data structure, preprocessing, feature regularization, and classifier choice. This design minimizes information leakage and provides a more reliable estimate of model performance compared with simplified preprocessing strategies. Simplified ablation analyses were not pursued because they could produce methodologically invalid comparisons or clinically implausible data representations.

A limitation of this study is the use of ultrasonography as the reference standard for hepatic steatosis. While ultrasonography is widely available and routinely used in clinical practice, its sensitivity for detecting mild steatosis is limited, particularly near the ≥5% threshold used in the current MASLD definition. As a result, some individuals with early steatosis may have been misclassified; however, such misclassification is expected to be largely non-differential and therefore more likely to attenuate, rather than inflate, model performance. This limitation may have particularly affected the distinction between mild steatosis and the absence of disease. Accordingly, the observed moderate yet consistent AUROC values should be interpreted as conservative estimates of the true predictive capability of the proposed models. Although the models demonstrated stable performance within the internal validation procedures, external validation in independent cohorts will be necessary to confirm generalizability across different populations and clinical settings. Importantly, this study was not intended to provide a definitive diagnostic alternative to advanced imaging modalities such as CAP or MRI-PDFF, but rather to develop a pragmatic and scalable pre-screening and risk stratification framework applicable in primary care and routine clinical settings to identify individuals who may benefit from confirmatory imaging or early preventive interventions. Accordingly, the proposed models should be interpreted as tools for prioritizing individuals for confirmatory imaging rather than as substitutes for ultrasonographic diagnosis.

Although deep learning architectures such as CNN- or transformer-based models may provide additional modeling flexibility, the present study focused on interpretable ML approaches using structured clinical data. Future studies with larger datasets may further explore deep learning methods to evaluate potential performance improvements.

Another consideration relates to the potential influence of medication use and temporal biological variability on model predictors. In the present cohort, glucose-lowering agents and statins were more frequently used among individuals with MASLD, reflecting the higher burden of metabolic comorbidities in this group. Such treatments, together with intra-individual fluctuations and transient metabolic states, may affect several biochemical parameters included in the model, including glucose levels, lipid profiles, and liver enzymes. Consequently, some predictors may partially reflect dynamic treatment-related or short-term metabolic conditions in addition to underlying disease biology. However, the study was intentionally designed to reflect real-world clinical conditions, where patients are commonly evaluated while receiving treatment for metabolic disorders. In this context, these factors represent part of the observable clinical phenotype rather than an artificial confounder to be fully removed. Nonetheless, future studies incorporating longitudinal measurements and treatment-stratified analyses are warranted to further clarify the relative contributions of stable hepatic pathology, metabolic variability, and therapeutic effects to model predictions.

This study was designed to maximize analytical repeatability and methodological transparency. All preprocessing, feature selection, and model training steps were implemented within a single, deterministic scikit-learn pipeline with fixed random seeds, ensuring that identical results can be reproduced when the same data and code are used. The use of repeated stratified cross-validation, standardized preprocessing procedures, and widely adopted open-source libraries further enhances repeatability and reduces susceptibility to implementation-specific variation.

Reproducibility across independent cohorts is supported by the exclusive use of routinely collected clinical, anthropometric, and biochemical variables, which are commonly available in electronic health records across healthcare systems. Although the underlying patient-level dataset cannot be publicly released due to ethical and institutional restrictions, the modeling workflow relies entirely on transparent, well-documented algorithms, allowing independent investigators to reproduce the analytical framework using their own datasets. To facilitate reproducibility, the analysis code and pipeline specifications can be made available upon reasonable request, enabling verification of results and extension of the methodology in external populations.

Future studies may further quantify the incremental contribution of individual preprocessing components through formal ablation and sensitivity analyses in independent external datasets.

## 5. Conclusions

Metabolic dysfunction-associated steatotic liver disease represents a substantial cardiometabolic disease burden, underscoring the need for practical and scalable approaches to the early identification of hepatic steatosis, particularly in primary care settings.

In this study, we demonstrate that supervised ML models developed exclusively from routinely collected clinical, anthropometric, and biochemical data can predict ultrasound-detected hepatic steatosis with moderate yet consistent and reproducible accuracy. Logistic regression and gradient boosting achieved the most favorable balance between predictive performance and clinical interpretability, while Elastic Net-based feature selection and SHAP analyses confirmed that model outputs were driven by biologically plausible and clinically meaningful variables.

When evaluated alongside established rule-based steatosis indices, the proposed ML models provided complete cohort coverage without indeterminate classifications, addressing a key practical limitation of commonly used screening scores. Although the absolute gain in discrimination is modest, its clinical relevance emerges from full applicability, balanced sensitivity–specificity trade-offs, and transparent model behavior.

Collectively, these findings support the potential of the proposed models to function as low-cost, automated pre-screening tools that can be seamlessly integrated into routine clinical workflows. By facilitating the early identification of individuals at increased risk who may benefit from confirmatory imaging or timely lifestyle interventions, this approach may support more rational allocation of healthcare resources. With further external and prospective validation, the proposed framework may provide a scalable and clinically implementable strategy for risk stratification of ultrasound-detected hepatic steatosis within the MASLD spectrum using routinely available clinical data in real-world practice.

## Figures and Tables

**Table 1:** Baseline demographic, anthropometric, clinical, and biochemical characteristics of participants according to ultrasonographic hepatic steatosis status.

Features	Normal USG	Hepatic Steatosis on USG	*p*-Value
*n* (%)	322 (50)	322 (50)	-
***Demographic and Anthropometric Characteristics***			
Age	62.36 ± 19.12 [60.26–64.46]	59.81 ± 15.95 [58.06–61.56]	0.004 *
Sex (Female)	179 (55.59)	170 (52.80)	0.477 †
Educational Status	57 (17.70)	57 (17.70)	1.000 †
Smoking	131 (40.68)	144 (44.72)	0.300 †
Height (cm)	164.19 ± 10.18 [163.08–165.31]	165.91 ± 9.98 [164.82–167.01]	0.043 *
Weight (kg)	70.43 ± 14.58 [68.83–72.03]	79.82 ± 18.24 [77.82–81.82]	<0.001 *
WC (cm)	87.71 ± 17.17 [85.83–89.59]	95.65 ± 18.24 [93.65–97.65]	<0.001 *
BMI	26.24 ± 5.77 [25.61–26.87]	29.02 ± 6.57 [28.30–29.74]	<0.001 *
Weekly Exercise History	71 (22.05)	44 (13.66)	**0.005** †
***Hemodynamic Parameters***			
SBP (mmHg)	124.29 ± 16.14 [122.52–126.06]	126.64 ± 17.28 [124.75–128.54]	0.026 *
DBP (mmHg)	72.63 ± 9.73 [71.56–73.69]	75.15 ± 11.10 [73.93–76.37]	<0.001 *
HR (beats/min)	80.35 ± 12.87 [78.94–81.76]	82.67 ± 13.16 [81.23–84.11]	0.031 *
***Clinical Comorbidities and Medication Use***			
DM	116 (36.02)	165 (51.24)	**<0.001** †
HTN	157 (48.76)	175 (54.35)	0.156 †
DLP	59 (18.32)	103 (31.99)	**<0.001** †
ASCVD	73 (22.67)	82 (25.47)	0.407 †
CVD	14 (4.35)	17 (5.28)	0.581 †
PCOS (in females)	1 (0.56)	2 (1.18)	0.563 †
OSA Syndrome	0 (0)	5 (1.55)	**0.025** †
Metformin Use	40 (12.42)	83 (25.78)	**<0.001** †
Pioglitazone Use	3 (0.93)	4 (1.24)	0.704 †
SGLT-2i Use	26 (8.07)	45 (13.98)	**0.017** †
Statin Use	39 (12.11)	65 (20.19)	**0.005** †
***Hematologic Parameters***			
Hb (g/dL)	11.12 ± 2.56 [10.84–11.40]	11.81 ± 2.61 [11.52–12.09]	**<0.001 ‡**
WBC (10^3^/µL)	8.41 ± 3.6 [8.01–8.80]	8.82 ± 3.52 [8.43–9.20]	0.110 *
Lymphocyte Count (10^3^/µL)	2.16 ± 3.04 [1.83–2.50]	2.10 ± 1.52 [1.93–2.27]	0.004 *
Neutrophil Count (10^3^/µL)	5.83 ± 3.34 [5.46–6.19]	5.96 ± 3.35 [5.59–6.33]	0.630 *
Monocyte Count (10^3^/µL)	0.74 ± 0.78 [0.66–0.83]	0.79 ± 0.84 [0.69–0.88]	0.339 *
Platelet Count (10^3^/µL)	253.55 ± 113.88 [241.07–266.04]	262.55 ± 103.84 [251.16–273.93]	0.290 *
***Biochemical Parameters***			
Fasting Plasma Glucose (mg/dL)	126.06 ± 63.27 [119.12–133]	144.47 ± 81.20 [135.57–153.37]	<0.001 *
BUN (mg/dL)	28.06 ± 23.42 [25.49–30.63]	27.79 ± 23.97 [25.16–30.42]	0.710 *
Cr (mg/dL)	1.28 ± 1.08 [1.16–1.40]	1.19 ± 0.80 [1.10–1.28]	0.658 *
eGFR (mL/min/1.73 m^2^)	70.74 ± 34.64 [66.94–74.54]	73.12 ± 33.29 [69.47–76.77]	0.287 *
Total Cholesterol (mg/dL)	152.09 ± 52.52 [146.34–157.85]	159.67 ± 54.58 [153.69–165.66]	0.039 *
LDL-C (mg/dL)	91.07 ± 39.71 [86.72–95.42]	94.37 ± 40.61 [89.92–98.82]	0.271 *
HDL-C (mg/dL)	38.64 ± 14.27 [37.07–40.20]	37.32 ± 13.71 [35.81–38.82]	0.202 *
TG (mg/dL)	134.36 ± 153.57 [117.53–151.20]	170.91 ± 116.66 [158.12–183.70]	<0.001 *
AST (U/L)	38.76 ± 87.87 [29.13–48.40]	43.94 ± 93.24 [33.71–54.16]	0.198 *
ALT (U/L)	40.60 ± 98.78 [29.77–51.43]	39.87 ± 78.22 [31.30–48.45]	<0.001 *
GGT (U/L)	66.91 ± 106.53 [55.23–78.59]	88.30 ± 175.22 [69.09–107.51]	0.167 *
HbA1c (%)	6.51 ± 2.34 [6.26–6.77]	7.11 ± 2.78 [6.81–7.41]	<0.001 *
Albumin (g/L)	36.03 ± 5.05 [35.47–36.58]	37.69 ± 4.56 [37.19–38.19]	<0.001 *
Direct Bilirubin (mg/dL)	0.30 ± 0.64 [0.23–0.37]	0.31 ± 0.56 [0.25–0.37]	0.714 *
Indirect Bilirubin (mg/dL)	0.35 ± 0.27 [0.32–0.38]	0.40 ± 0.45 [0.35–0.45]	0.488 *
TSH (mIU/L)	2.02 ± 3.10 [1.68–2.36]	2.65 ± 6.72 [1.91–3.38]	0.120 *
FT4 (ng/dL)	1.26 ± 0.27 [1.23–1.29]	1.24 ± 0.23 [1.21–1.26]	0.490 *
UA (mg/dL)	5.53 ± 2.06 [5.30–5.75]	5.75 ± 1.79 [5.55–5.94]	0.096 *
Ferritin (μg/L)	221.65 ± 251.39 [194.09–249.21]	241.63 ± 305.89 [208.10–275.17]	0.227 *
Vitamin B12 (ng/L)	448.49 ± 294.90 [416.16–480.82]	445.07 ± 269.07 [415.57–474.57]	0.199 *
ALP (U/L)	111.11 ± 96.62 [100.51–121.70]	107.58 ± 92.81 [97.41–117.76]	0.561 *

Abbreviations: USG: Ultrasonography; BMI: Body mass index; SBP: Systolic blood pressure; DBP: Diastolic blood pressure; HR: Heart rate; DM: Diabetes mellitus; HTN: Hypertension; DLP: Dyslipidemia; ASCVD: Atherosclerotic cardiovascular disease; CVD: Cerebrovascular disease; PCOS: Polycystic ovary syndrome; OSA: Obstructive sleep apnea; SGLT-2i: Sodium–glucose cotransporter-2 inhibitor; Hb: Hemoglobin; WBC: White blood cell; AST: Aspartate aminotransferase; ALT: Alanine aminotransferase; GGT: Gamma-glutamyl transferase; BUN: Blood urea nitrogen; Cr: Creatinine; eGFR: Estimated glomerular filtration rate; LDL-C: Low-density lipoprotein cholesterol; HDL-C: High-density lipoprotein cholesterol; TG: Triglycerides; HbA1c: Glycated hemoglobin; TSH: Thyroid-stimulating hormone; FT4: Free thyroxine; UA: Uric acid; ALP: Alkaline phosphatase; WC: Waist circumference; *: Mann–Whitney U test; †: Chi-square test; ‡: Student’s *t*-test. Bold values indicate statistically significant results. Bold and italic formatting were used to distinguish subsection headings within the table.

**Table 2:** Comparison of derived body composition, metabolic, cardiovascular, hepatic, hematologic, and renal indices between participants with and without ultrasonographic hepatic steatosis.

Features	Normal USG	Hepatic Steatosis on USG	*p*-Value
*n* (%)	322 (50)	322 (50)	-
***Body Composition Indices***			
WtHR	0.54 ± 0.11 [0.52–0.55]	0.58 ± 0.11 [0.57–0.59]	<0.001 *
ABSI	0.08 ± 0.01 [0.08–0.08]	0.08 ± 0.01 [0.08–0.08]	0.074 *
Body Fat Percentage	35.63 ± 10.98 [34.43–36.84]	38.08 ± 11.74 [36.80–39.37]	**0.006 ‡**
PI	16.13 ± 4.43 [15.65–16.62]	17.59 ± 4.39 [17.11–18.07]	<0.001 *
CI	1.23 ± 0.17 [1.21–1.25]	1.27 ± 0.16 [1.25–1.28]	**0.004 ‡**
RFM	31.84 ± 10.38 [30.70–32.97]	34.40 ± 10.04 [33.30–35.50]	**0.002 ‡**
***Metabolic Indices***			
TyG	8.76 ± 0.75 [8.68–8.84]	9.14 ± 0.81 [9.05–9.22]	**<0.001 ‡**
TyG/HDL Ratio	4.73 ± 11.37 [3.49–5.98]	5.60 ± 5.85 [4.96–6.24]	<0.001 *
AIP	0.12 ± 0.34 [0.09–0.16]	0.25 ± 0.33 [0.22–0.29]	**<0.001 ‡**
LAP	43.90 ± 84.95 [34.59–53.22]	68.88 ± 62.30 [62.05–75.71]	<0.001 *
VAI	3.31 ± 7.41 [2.50–4.12]	3.95 ± 4.20 [3.49–4.41]	<0.001 *
TyG-BMI	230.16 ± 55.79 [224.05–236.28]	266.41 ± 71.26 [258.60–274.22]	<0.001 *
TyG-WC	769.58 ± 171.00 [750.83–788.33]	876.31 ± 195.00 [854.93–897.69]	**<0.001 ‡**
TyG-WHtR	4.70 ± 1.08 [4.58–4.82]	5.29 ± 1.20 [5.16–5.42]	<0.001 *
***Cardiovascular Indices***			
Castelli I	4.41 ± 2.68 [4.12–4.70]	4.70 ± 2.26 [4.46–4.95]	0.004 *
Castelli II	2.55 ± 1.38 [2.40–2.70]	2.75 ± 1.33 [2.60–2.89]	0.047 *
Non-HDL-C	113.46 ± 49.47 [108.03–118.88]	122.36 ± 52.14 [116.64–128.07]	0.020 *
RC	22.39 ± 30.81 [19.01–25.77]	27.99 ± 26.75 [25.06–30.92]	<0.001 *
PP	51.66 ± 12.98 [50.24–53.09]	51.49 ± 14.05 [49.95–53.03]	0.783 *
RPP	9977.79 ± 2025.54 [9755.71–10,199.87]	10,463.94 ± 2157.03 [10,227.45–10,700.43]	<0.001 *
***Liver Indices***			
De Ritis Ratio	1.25 ± 0.56 [1.19–1.32]	1.19 ± 0.63 [1.12–1.26]	0.006 *
APRI	0.77 ± 5.61 [0.16–1.39]	0.87 ± 5.69 [0.25–1.50]	0.893 *
FIB-4	2.23 ± 5.71 [1.60–2.85]	2.43 ± 8.33 [1.52–3.35]	0.088 *
HSI	36.08 ± 8.07 [35.19–36.96]	39.41 ± 7.95 [38.53–40.28]	**<0.001 ‡**
NFS	−0.93 ± 1.99 [−1.15–−0.71]	−0.88 ± 2.06 [−1.11–−0.65]	0.768 **‡**
ALBI	−2.41 ± 0.56 [−2.47–−2.35]	−2.51 ± 0.59 [−2.57–−2.44]	0.003 *
HALP	43.02 ± 54.53 [37.04–49.00]	44.43 ± 57.84 [38.09–50.77]	0.024 *
***Immune/Hematologic Scores***			
NLR	3.79 ± 2.90 [3.47–4.11]	3.64 ± 3.09 [3.30–3.98]	0.160 *
PLR	158.85 ± 96.20 [148.30–169.40]	148.72 ± 79.59 [139.99–157.44]	0.401 *
MLR	0.43 ± 0.27 [0.40–0.46]	0.45 ± 0.53 [0.39–0.50]	0.279 *
SII	985.99 ± 936.22 [883.35–1088.64]	962.46 ± 1118.44 [839.83–1085.08]	0.682 *
SIRI	2.80 ± 2.92 [2.48–3.12]	2.94 ± 3.67 [2.54–3.35]	0.644 *
PNI	46.84 ± 16.49 [45.04–48.65]	48.19 ± 9.37 [47.16–49.22]	<0.001 *
***Renal Indices***			
BUN/Cr Ratio	33.74 ± 71.53 [25.89–41.58]	29.68 ± 54.69 [23.68–35.67]	0.359 *
UHR	0.17 ± 0.12 [0.16–0.18]	0.18 ± 0.10 [0.17–0.19]	0.031 *
UA/Cr Ratio	6.50 ± 7.65 [5.66–7.34]	6.48 ± 6.05 [5.81–7.14]	0.055 *

Abbreviations: WHtR: Waist-to-height ratio; ABSI: A body shape index; PI: Ponderal index; CI: Conicity index; RFM: Relative fat mass; TyG: Triglyceride–glucose index; AIP: Atherogenic index of plasma; LAP: Lipid accumulation product; VAI: Visceral adiposity index; TyG-BMI: TyG-Body mass index; Castelli I: Total cholesterol to HDL-C ratio; Castelli II: LDL-C to HDL-C ratio; RC: Remnant cholesterol; PP: Pulse pressure; RPP: Rate pressure product; APRI: AST-to-platelet ratio index; FIB-4: Fibrosis-4 index; HSI: Hepatic steatosis index; NFS: Non-alcoholic fatty liver disease (NAFLD) fibrosis score; ALBI: Albumin–bilirubin score; HALP: Hemoglobin–albumin–lymphocyte–platelet score; NLR: Neutrophil-to-lymphocyte ratio; PLR: Platelet-to-lymphocyte ratio; MLR: Monocyte-to-lymphocyte ratio; SII: Systemic immune–inflammation index; SIRI: Systemic inflammation response index; PNI: Prognostic nutritional index; UHR: Uric acid-to-HDL-C ratio; *: Mann–Whitney U test; ‡: Student’s *t*-test; Remaining abbreviations are as defined in Table 1. Bold values indicate statistically significant results. Bold and italic formatting were used to distinguish subsection headings within the table.

**Table 3:** Comparative performance of machine learning classifiers for prediction of ultrasound-detected hepatic steatosis within the MASLD spectrum.

Model	Accuracy	Sensitivity	Specificity	NPV	PPV	F1 Score	Youden Index	ROC AUC
**Decision Tree**	0.5823 ± 0.0413 95% CI: 0.5652–0.5994	0.5852 ± 0.0736 95% CI: 0.5549–0.6156	0.5795 ± 0.0417 95% CI: 0.5623–0.5968	0.5851 ± 0.0461 95% CI: 0.5661–0.6041	0.5806 ± 0.0381 95% CI: 0.5649–0.5964	0.5819 ± 0.0533 95% CI: 0.5599–0.6039	0.1648 ± 0.0826 95% CI: 0.1307–0.1989	0.5824 ± 0.0413 95% CI: 0.5653–0.5994
**AdaBoost**	0.5764 ± 0.0420 95% CI: 0.5591–0.5938	0.5735 ± 0.0674 95% CI: 0.5457–0.6013	0.5796 ± 0.0457 95% CI: 0.5607–0.5984	0.5777 ± 0.0463 95% CI: 0.5586–0.5968	0.5763 ± 0.0397 95% CI: 0.5599–0.5927	0.5740 ± 0.0501 95% CI: 0.5533–0.5946	0.1531 ± 0.0838 95% CI: 0.1185–0.1877	0.5765 ± 0.0419 95% CI: 0.5592–0.5938
**Random Forest**	0.6336 ± 0.0417 95% CI: 0.6164–0.6508	0.6013 ± 0.0494 95% CI: 0.5809–0.6217	0.6659 ± 0.0702 95% CI: 0.6369–0.6949	0.6253 ± 0.0388 95% CI: 0.6093–0.6413	0.6457 ± 0.0518 95% CI: 0.6243–0.6670	0.6213 ± 0.0400 95% CI: 0.6047–0.6378	0.2672 ± 0.0834 95% CI: 0.2328–0.3017	0.6846 ± 0.0394 95% CI: 0.6683–0.7008
**XGBoost**	0.6264 ± 0.0303 95% CI: 0.6139–0.6389	0.6204 ± 0.0512 95% CI: 0.5993–0.6415	0.6324 ± 0.0554 95% CI: 0.6096–0.6553	0.6256 ± 0.0303 95% CI: 0.6131–0.6381	0.6291 ± 0.0351 95% CI: 0.6147–0.6436	0.6235 ± 0.0343 95% CI: 0.6094–0.6377	0.2528 ± 0.0605 95% CI: 0.2279–0.2778	0.6740 ± 0.0269 95% CI: 0.6629–0.6851
**Gradient Boosting**	0.6479 ± 0.0340 95% CI: 0.6338–0.6619	0.6443 ± 0.0559 95% CI: 0.6213–0.6674	0.6517 ± 0.0591 95% CI: 0.6273–0.6761	0.6479 ± 0.0368 95% CI: 0.6327–0.6631	0.6506 ± 0.0382 95% CI: 0.6348–0.6664	0.6460 ± 0.0366 95% CI: 0.6309–0.6611	0.2960 ± 0.0678 95% CI: 0.2681–0.3240	0.6824 ± 0.0383 95% CI: 0.6666–0.6983
**SVM**	0.6267 ± 0.0356 95% CI: 0.6120–0.6414	0.5665 ± 0.0436 95% CI: 0.5485–0.5844	0.6870 ± 0.0565 95% CI: 0.6637–0.7103	0.6130 ± 0.0312 95% CI: 0.6001–0.6259	0.6459 ± 0.0453 95% CI: 0.6272–0.6646	0.6026 ± 0.0370 95% CI: 0.5873–0.6179	0.2535 ± 0.0713 95% CI: 0.2240–0.2829	0.6783 ± 0.0427 95% CI: 0.6607–0.6959
**KNN**	0.5916 ± 0.0347 95% CI: 0.5773–0.6059	0.5939 ± 0.0617 95% CI: 0.5685–0.6194	0.5895 ± 0.0551 95% CI: 0.5668–0.6123	0.5931 ± 0.0376 95% CI: 0.5776–0.6086	0.5916 ± 0.0343 95% CI: 0.5774–0.6057	0.5915 ± 0.0421 95% CI: 0.5741–0.6089	0.1835 ± 0.0693 95% CI: 0.1549–0.2121	0.6207 ± 0.0369 95% CI: 0.6055–0.6360
**MLP**	0.6137 ± 0.0367 95% CI: 0.5985–0.6288	0.5994 ± 0.0582 95% CI: 0.5754–0.6234	0.6280 ± 0.0512 95% CI: 0.6068–0.6491	0.6115 ± 0.0373 95% CI: 0.5962–0.6269	0.6174 ± 0.0392 95% CI: 0.6013–0.6336	0.6072 ± 0.0429 95% CI: 0.5895–0.6249	0.2274 ± 0.0731 95% CI: 0.1972–0.2575	0.6558 ± 0.0389 95% CI: 0.6397–0.6718
**Naive Bayes**	0.5767 ± 0.0583 95% CI: 0.5527–0.6008	0.6510 ± 0.2014 95% CI: 0.5678–0.7341	0.5032 ± 0.2168 95% CI: 0.4137–0.5927	0.5929 ± 0.0866 95% CI: 0.5571–0.6286	0.5769 ± 0.0568 95% CI: 0.5534–0.6003	0.5905 ± 0.1088 95% CI: 0.5456–0.6354	0.1541 ± 0.1159 95% CI: 0.1063–0.2020	0.6326 ± 0.0550 95% CI: 0.6098–0.6553
**Logistic Regression**	0.6516 ± 0.0347 95% CI: 0.6373–0.6659	0.6379 ± 0.0577 95% CI: 0.6141–0.6617	0.6653 ± 0.0641 95% CI: 0.6389–0.6918	0.6485 ± 0.0340 95% CI: 0.6345–0.6626	0.6578 ± 0.0419 95% CI: 0.6406–0.6751	0.6460 ± 0.0387 95% CI: 0.6301–0.6620	0.3033 ± 0.0694 95% CI: 0.2746–0.3319	0.7148 ± 0.0399 95% CI: 0.6983–0.7312

Abbreviations: SVM: Support vector machine; MLP: Multilayer perceptron; KNN: k-nearest neighbors; NPV: Negative predictive value; PPV: Positive predictive value; ROC AUC: Area under the receiver operating characteristic curve. Bold formatting was used to improve the readability of the table and to facilitate clearer distinction of the subsection headings.

**Table 4:** Comparative performance and clinical characteristics of machine-learning models versus steatosis indices.

Approach	Output Type	Coverage of Cohort	Sensitivity	Specificity	Accuracy	ROC AUC	Key Clinical Characteristics
**Logistic Regression (ML)**	Continuous probability	100% (644/644) Indeterminate cases: 0%	0.64	0.67	0.65	0.71	Balanced discrimination; interpretable coefficients; no indeterminate zone; suitable for automated screening
**Gradient Boosting (ML)**	Continuous probability	100% (644/644) Indeterminate cases: 0%	0.65	0.65	0.65	0.68	Nonlinear modeling; stable performance; full cohort applicability
**HSI** **(rule-based)**	Binary decision rule (rule-in/rule-out)	71.30% (459/644) Indeterminate cases: 28.70%	0.84	0.42	0.63	0.63	Designed for rule-out; high sensitivity but low specificity; large indeterminate group
**FLI** **(rule-based)**	Binary decision rule (rule-in/rule-out)	76.60% (493/644) Indeterminate cases: 23.40%	0.71	0.55	0.63	0.63	Balanced rule-in/rule-out tool; moderate discrimination; indeterminate zone remains

Abbreviations: ML: Machine learning; FLI: Fatty liver index; Remaining abbreviations are as defined in Table 2 and Table 3. Notes: For ML models, sensitivity, specificity, accuracy, and balanced accuracy were calculated at a fixed probability threshold of 0.5, whereas AUROC was derived from continuous predicted probabilities. Indeterminate cases were excluded only for rule-based indices (HSI and FLI). Bold formatting was used to improve the readability of the table and to facilitate clearer distinction of the subsection headings.

## Data Availability

The data presented in this study are available on reasonable request from the corresponding author due to ethical and institutional restrictions.

## Supplementary Materials

The following supporting information can be downloaded at: https://www.mdpi.com/article/10.3390/biomedicines14051154/s1. Figure S1. Elastic Net–based variable importance for ultrasound-detected hepatic steatosis within the MASLD spectrum. Figure S2. Elastic Net—Top 20 features ranked by the absolute value of their coefficients (|β|).

## Abbreviations

The following abbreviations are used in this manuscript:

MASLD	Metabolic dysfunction-associated steatotic liver disease
ML	Machine learning
CAP	Controlled attenuation parameter
MRI-PDFF	Magnetic resonance imaging-proton density fat fraction
AUROC	Area under the Receiver Operating Characteristic Curve
SHAP	Shapley Additive Explanations
BMI	Body mass index
FIB-4	Fibrosis-4 index
BUN	Blood urea nitrogen
Cr	Creatinine
APRI	Aspartate aminotransferase to platelet ratio index
FLI	Fatty liver index
HSI	Hepatic steatosis index
SAGA	Stochastic Average Gradient Augmented
SAMME	Stagewise Additive Modeling using a Multiclass Exponential loss function
ROC	Receiver Operating Characteristic
PPV	Positive predictive value
NPV	Negative predictive value
LDL	Low-density lipoprotein
HDL	High-density lipoprotein
eGFR	Estimated glomerular filtration rate
AST	Aspartate aminotransferase
ALT	Alanine aminotransferase
ABSI	A body shape index
NAFLD	Nonalcoholic fatty liver disease
UA	Uric acid
WC	Waist circumference
VAI	Visceral adiposity index
TyG-WC	Triglyceride–glucose index adjusted for waist circumference
TyG-BMI	Triglyceride–glucose index adjusted for BMI
SVM	Support Vector Machine
MLP	Multilayer Perceptron
DL	Deep learning
SLD	Steatotic liver disease
IGFBP7	Insulin-like growth factor binding protein 7
SSc5D	Scavenger receptor cysteine-rich Type 5 domain-containing protein
Sema4D	Semaphorin 4D
LightGBM	Light Gradient Boosting Machine
EHR	Electronic health record
HbA1c	Glycated hemoglobin
USG	Ultrasonography
SBP	Systolic blood pressure
DBP	Diastolic blood pressure
HR	Heart rate
DM	Diabetes mellitus
HTN	Hypertension
DLP	Dyslipidemia
ASCVD	Atherosclerotic cardiovascular disease
CVD	Cerebrovascular disease
PCOS	Polycystic ovary syndrome
OSA	Obstructive sleep apnea
SGLT-2i	Sodium–glucose cotransporter-2 inhibitor
Hb	Hemoglobin
WBC	White blood cell
GGT	Gamma-glutamyl transferase
ALP	Alkaline phosphatase
TG	Triglycerides
TSH	Thyroid-stimulating hormone
FT4	Free thyroxine
WHtR	Waist-to-height ratio
PI	Ponderal index
CI	Conicity index
RFM	Relative fat mass
AIP	Atherogenic index of plasma
LAP	Lipid accumulation product
Castelli I	Total cholesterol-to-HDL-cholesterol ratio
Castelli II	LDL-cholesterol-to-HDL-cholesterol ratio
RC	Remnant cholesterol
PP	Pulse pressure
RPP	Rate pressure product
NFS	NAFLD fibrosis score
ALBI	Albumin–bilirubin score
HALP	Hemoglobin–albumin–lymphocyte–platelet score
NLR	Neutrophil-to-lymphocyte ratio
PLR	Platelet-to-lymphocyte ratio
MLR	Monocyte-to-lymphocyte ratio
SII	Systemic immune-inflammation index
SIRI	Systemic inflammation response index
PNI	Prognostic nutritional index
UHR	Uric acid-to-HDL-cholesterol
KNN	k-nearest neighbors

## References

[1] Chan W., Chuah K., Rajaram R., Lim L., Ratnasingam J., Vethakkan S. (2023). Metabolic Dysfunction-Associated Steatotic Liver Disease (MASLD): A State-of-the-Art Review. J. Obes. Metab. Syndr..

[2] Li Y., Yang P., Ye J., Xu Q., Wu J., Wang Y. (2024). Updated mechanisms of MASLD pathogenesis. Lipids Health Dis..

[3] Hong S., Sun L., Hao Y., Li P., Zhou Y., Liang X., Hu J., Wei H. (2024). From NAFLD to MASLD: When metabolic comorbidity matters. Ann. Hepatol..

[4] Zazueta A., Valenzuela-Perez L., Ortiz-Lopez N., Pinto-Leon A., Torres V., Guinez D., Aliaga N., Merino P., Sandoval A., Covarrubias N. (2024). Alteration of Gut Microbiota Composition in the Progression of Liver Damage in Patients with Metabolic Dysfunction-Associated Steatotic Liver Disease (MASLD). Int. J. Mol. Sci..

[5] Fan J., Xu X., Yang R., Nan Y., Wei L., Jia J., Zhuang H., Shi J., Li X., Sun C. (2024). Guideline for the Prevention and Treatment of Metabolic Dysfunction-associated Fatty Liver Disease (Version 2024). J. Clin. Transl. Hepatol..

[6] Lu H., Mao Z., Zheng M., Zhang M., Huang H., Chen Y., Lv L., Chen Z. (2025). Identification of hub gene for the pathogenic mechanism and diagnosis of MASLD by enhanced bioinformatics analysis and machine learning. PLoS ONE.

[7] DiBattista J., Burkholder D., Lok A., Chen V. (2022). Accuracy of Non-invasive Indices for Diagnosing Hepatic Steatosis Compared to Imaging in a Real-World Cohort. Dig. Dis. Sci..

[8] Wu J., Li H., Xu Z., Ran L., Kong L. (2021). Population-specific cut-off points of fatty liver index for the diagnosis of hepatic steatosis. J. Hepatol..

[9] Su P., Chen Y., Lin C., Su W., Huang S., Yen H. (2023). Comparison of Machine Learning Models and the Fatty Liver Index in Predicting Lean Fatty Liver. Diagnostics.

[10] Frey L., Fuchs M., Ward R., Gebregziabher M., Nasir A., Natarajan Y., Schreiner A., Rockey D., Syn W. (2025). Use of machine learning for early prediction of short-term mortality in veterans with metabolic dysfunction-associated steatotic liver disease. PLoS ONE.

[11] Chen H., Zhang J., Chen X., Luo L., Dong W., Wang Y., Zhou J., Chen C., Wang W., Zhang W. (2025). Development and validation of machine learning models for MASLD: Based on multiple potential screening indicators. Front. Endocrinol..

[12] Soliman R., Helmy A., Shiha G. (2025). Precision in Diagnosis of Liver Fibrosis in MASLD: Machine Learning-Based Scores May Be More Accurate Than Conventional NITs. Liver Int..

[13] Weng S., Hu D., Chen J., Yang Y., Peng D. (2023). Prediction of fatty liver disease in a Chinese population using machine-learning algorithms. Diagnostics.

[14] Marques R., Santos J., André A., Silva J. (2024). Ultrasound versus elastography in the diagnosis of hepatic steatosis: Evaluation of traditional machine learning versus deep learning. Sensors.

[15] Mahzari A. (2022). Artificial intelligence in nonalcoholic fatty liver disease. Egypt. Liver J..

[16] Kirik A., Sumbul H.E., Koca N., Paşalı Kilit T., Demiral Sezer S., Binnetoglu E., Araç E., Solmaz İ., Şen H., Demirci İ. (2025). Prevalence of MASLD and fibrosis risk in Turkish adults with cardiometabolic risk factors: A nationwide multicenter study (DAHUDER MASLD study). J. Clin. Med..

[17] Bedogni G., Bellentani S., Miglioli L., Masutti F., Passalacqua M., Castiglione A., Tiribelli C. (2006). The Fatty Liver Index: A simple and accurate predictor of hepatic steatosis in the general population. BMC Gastroenterol..

[18] Lee J., Kim D., Kim H., Lee C., Yang J., Kim W., Kim Y., Yoon J., Cho S., Sung M. (2010). Hepatic steatosis index: A simple screening tool reflecting nonalcoholic fatty liver disease. Dig. Liver Dis..

[19] Huang D., El-Serag H., Loomba R. (2021). Global epidemiology of NAFLD-related HCC: Trends, predictions, risk factors and prevention. Nat. Rev. Gastroenterol. Hepatol..

[20] Lazarus J., Mark H., Allen A., Arab J., Carrieri P., Noureddin M., Alazawi W., Alkhouri N., Alqahtani S., Anstee Q. (2024). A global action agenda for turning the tide on fatty liver disease. Hepatology.

[21] Crudele L., De Matteis C., Novielli F., Di Buduo E., Petruzzelli S., De Giorgi A., Antonica G., Berardi E., Moschetta A. (2024). Fatty Liver Index (FLI) is the best score to predict MASLD with 50% lower cut-off value in women than in men. Biol. Sex Differ..

[22] McTeer M., Applegate D., Mesenbrink P., Ratziu V., Schattenberg J.M., Bugianesi E., Geier A., Romero Gomez M., Dufour J.-F., Ekstedt M. (2024). Machine learning approaches to enhance diagnosis and staging of patients with MASLD using routinely available clinical information. PLoS ONE.

[23] Cubillos G., Perez-Valenzuela J., Aguirre H., Martinez L., Castro L., Mezzano G., Perez C. (2025). Development of a novel deep learning method that transforms tabular input variables into images for the prediction of SLD. Sci. Rep..

[24] Lim D., Chung G., Cher P., Chockalingam R.J., Kim W., Tan C. (2024). Use of Machine Learning to Predict Onset of NAFLD in an All-Comers Cohort-Development and Validation in 2 Large Asian Cohorts. Gastro Hep Adv..

[25] Fabbrini E., Sullivan S., Klein S. (2010). Obesity and Nonalcoholic Fatty Liver Disease: Biochemical, Metabolic, and Clinical Implications. Hepatology.

[26] Demirci S., Sezer S. (2025). Fatty Liver Index vs. Biochemical-Anthropometric Indices: Diagnosing Metabolic Dysfunction-Associated Steatotic Liver Disease with Non-Invasive Tools. Diagnostics.

[27] Qian X., Wu W., Chen B., Zhang S., Xiao C., Chen L., Chen J., Ke L., He M., Li X. (2025). Value of triglyceride glucose-body mass index in predicting nonalcoholic fatty liver disease in individuals with type 2 diabetes mellitus. Front. Endocrinol..

[28] Sheng G., Lu S., Xie Q., Peng N., Kuang M., Zou Y. (2021). The usefulness of obesity and lipid-related indices to predict the presence of Non-alcoholic fatty liver disease. Lipids Health Dis..

[29] Xuan Y., Wu D., Zhang Q., Yu Z., Yu J., Zhou D. (2024). Elevated ALT/AST ratio as a marker for NAFLD risk and severity: Insights from a cross-sectional analysis in the United States. Front. Endocrinol..

[30] Rigor J., Diegues A., Presa J., Barata P., Martins-Mendes D. (2022). Noninvasive fibrosis tools in NAFLD: Validation of APRI, BARD, FIB-4, NAFLD fibrosis score, and Hepamet fibrosis score in a Portuguese population. Postgrad. Med..

[31] Ouzan D., Penaranda G., Jlaiel M., Joly H., Corneille J. (2024). Using the FIB-4, automatically calculated, followed by the ELF test in second line to screen primary care patients for liver disease. Sci. Rep..

[32] Yang C., He Q., Chen Z., Qin J., Lei F., Liu Y., Liu W., Chen M., Sun T., Zhu Q. (2022). A Bidirectional Relationship Between Hyperuricemia and Metabolic Dysfunction-Associated Fatty Liver Disease. Front. Endocrinol..

[33] Francoz C., Durand F., Kahn J., Genyk Y., Nadim M. (2019). Hepatorenal Syndrome. Clin. J. Am. Soc. Nephrol..

[34] Bucurica S., Nancoff A., Dutu M., Mititelu M., Gaman L., Ionia-Radu F., Jinga M., Maniu I., Ruta F. (2024). Exploring the Relationship between Lipid Profile, Inflammatory State and 25-OH Vitamin D Serum Levels in Hospitalized Patients. Biomedicines.

[35] Belalcazar S., Acosta E., Medina-Murillo J., Salcedo-Cifuentes M. (2020). Conventional biomarkers for cardiovascular risks and their correlation with the castelli risk index-indices and TG/HDL-c. Arch. Med..

[36] Xiao L., Zeng L., Wang J., Hong C., Zhang Z., Wu C., Cui H., Li Y., Li R., Liang S. (2025). Development and Validation of Machine Learning-Based Marker for Early Detection and Prognosis Stratification of Nonalcoholic Fatty Liver Disease. Adv. Sci..

[37] Verschuren L., Mak A., van Koppen A., Oezsezen S., Difrancesco S., Caspers M., Snabel J., van der Meer D., van Dijk A., Rashu E. (2024). Development of a novel non-invasive biomarker panel for hepatic fibrosis in MASLD. Nat. Commun..

[38] Yu Y., Yang Y., Li Q., Yuan J., Zha Y. (2025). Predicting metabolic dysfunction associated steatotic liver disease using explainable machine learning methods. Sci. Rep..

[39] Koliaki C., Dalamaga M., Kakounis K., Liatis S. (2025). Metabolically Healthy Obesity and Metabolic Dysfunction-Associated Steatotic Liver Disease (MASLD): Navigating the Controversies in Disease Development and Progression. Curr. Obes. Rep..

[40] Collins G., Moons K., Dhiman P., Riley R., Beam A., Van Calster B., Ghassemi M., Liu X., Reitsma J., van Smeden M. (2024). TRIPOD plus AI statement: Updated guidance for reporting clinical prediction models that use regression or machine learning methods. BMJ-Br. Med. J..

