# Supplementary material for: Machine Learning-Based Prediction of Ultrasound-Detected Hepatic Steatosis Within the Metabolic Dysfunction-Associated Steatotic Liver Disease Spectrum Using Routine Clinical and Biochemical Parameters
Source: Biomedicines. 2026 May 20;14(5):1154. doi: 10.3390/biomedicines14051154 (PMC13204908; doi:10.3390/biomedicines14051154)
Supplement: Supplementary file 1 [file biomedicines-14-01154-s001.zip › biomedicines-4270791-supplementary.pdf]

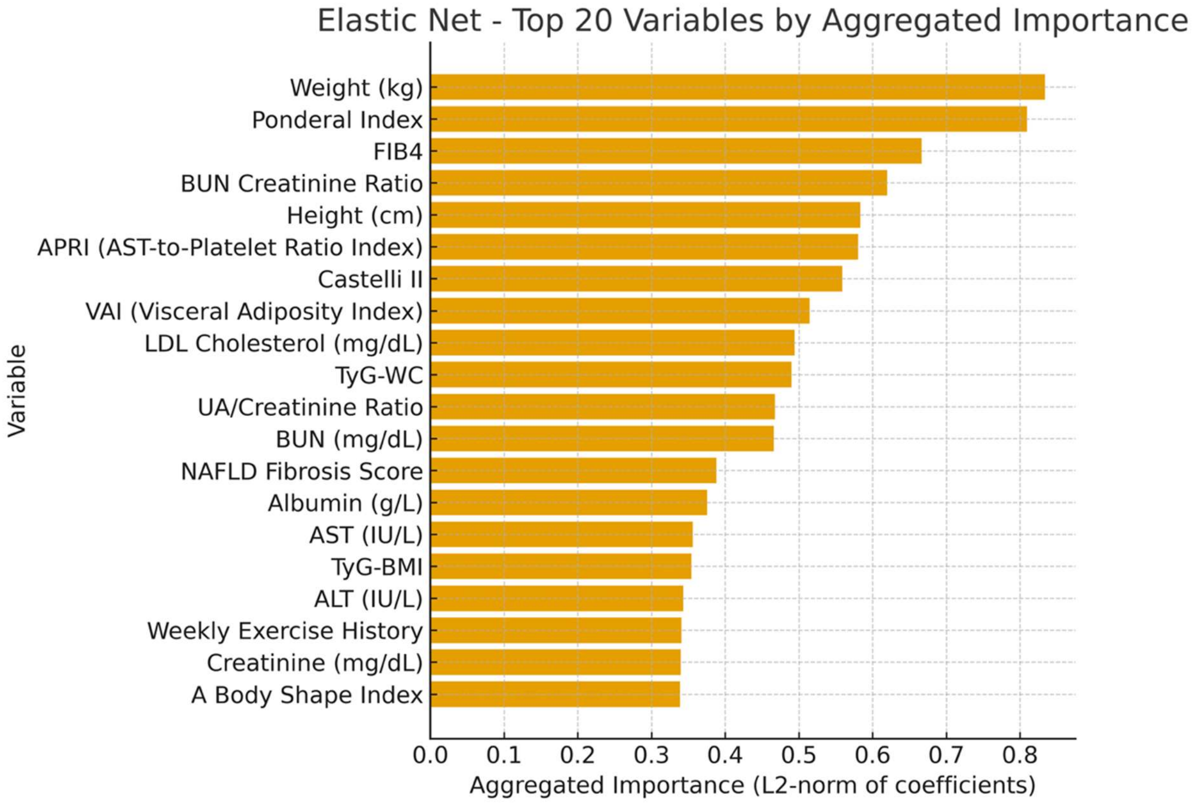

Supplementary Figure-1. Elastic Net–based variable importance for ultrasound-detected hepatic steatosis within the MASLD spectrum. Horizontal bars show the aggregated importance of the top 20 variables, calculated as the L2 norm of all coefficients corresponding to each original variable after one-hot encoding. Variables are ordered from highest to lowest importance; bar length reflects magnitude only (direction/sign is not shown). Preprocessing included median imputation and z-score standardization for numeric variables, and mode imputation with one-hot encoding for categorical variables. The model was a logistic regression with Elastic Net regularization fitted using the SAGA solver. (Abbreviations: Abbreviations are as defined in Table 1 and in Table 2.)

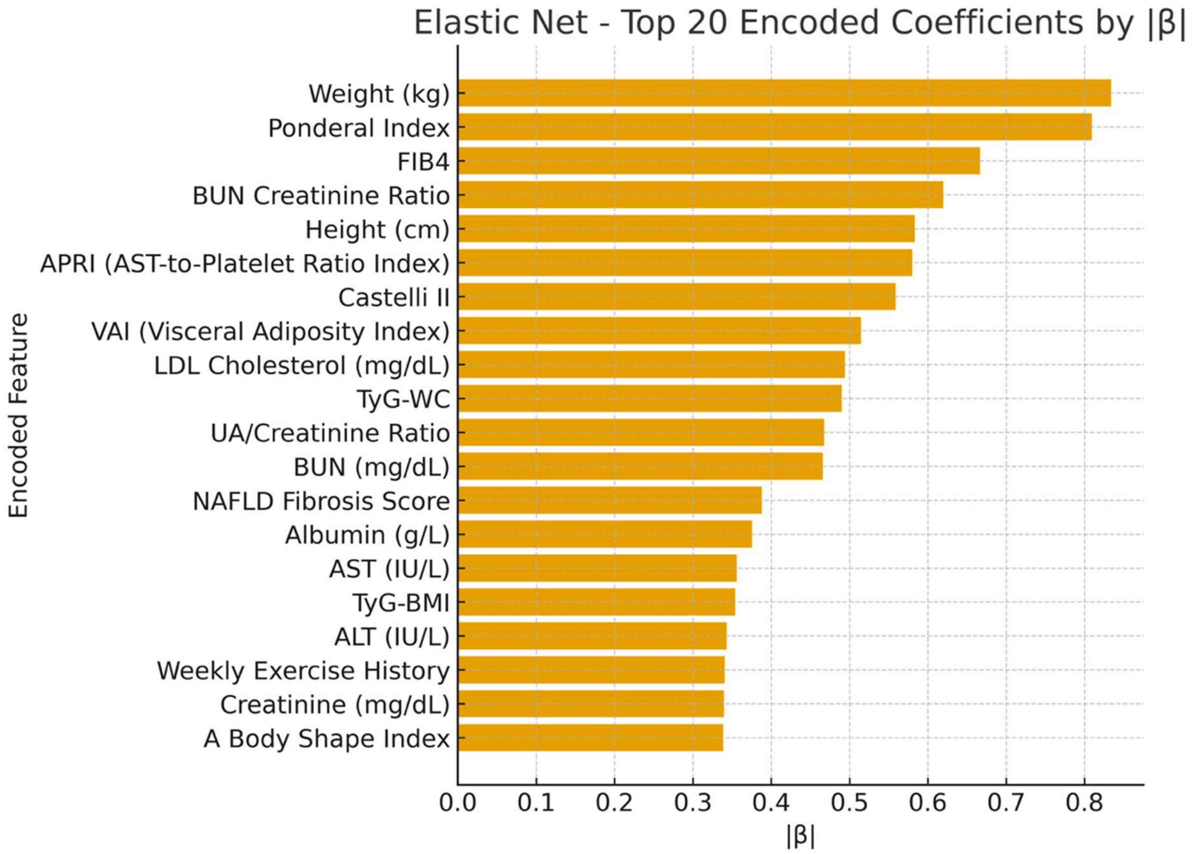

Supplementary Figure 2. Elastic Net—Top 20 features ranked by the absolute value of their coefficients (|β|). Horizontal bars represent the absolute magnitude of the coefficients from the fitted Elastic Net logistic regression model. Variables are ordered from highest to lowest |β|. Preprocessing and model specification were identical to those described in Supplementary Figure S1. Abbreviations are as defined in Table 1. (Abbreviations: |β|, absolute value of the coefficient; Remaining abbreviations are as defined in Table 1 and in Table 2).
